# Evaluating the impact of exercise on intermediate disease markers in overweight and obese individuals through a network meta-analysis of randomized controlled trials

**DOI:** 10.1038/s41598-024-62677-w

**Published:** 2024-05-27

**Authors:** Yuanyuan Liu, Xianzi Wang, Zilong Fang

**Affiliations:** 1 School of Sports Medicine and Rehabilitation, Beijing Sport University, Haidian District, Beijing, 100084 China; 2 School of Computer Science, Anhui University of Technology, Huashan District, Ma’anshan City, 243002 China

**Keywords:** Exercise, Overweight and obesity, Intermediate disease markers, Network meta-analysis, Biomarkers, Health care

## Abstract

The aim of this study is to investigate the impact of exercise on intermediate disease markers in populations with overweight and obesity, providing evidence-based recommendations for clinicians to utilize these markers in developing exercise prescriptions for this group. The study was conducted by retrieving data from PubMed, Embase, Cochrane Library, Web of Science, and CNKI and only including Randomized Controlled Trials (RCTs) to examine the effect of different exercise interventions on intermediate disease markers in overweight and obese people. The quality of the included studies was evaluated using the Cochrane Bias Risk Assessment tool and the data was analyzed using Stata 15.1 data analysis software. The RCTs were collected from January 2017 to March 2024. A total of 56 RCTs were included and the results of 10 outcomes were analyzed using random effects meta-analysis. The total sample size used in the study was 3193 The results showed that resistance training significantly reduced total cholesterol (SUCRA: 99.9%), triglycerides (SUCRA: 100.0%), low-density lipoprotein (SUCRA: 100.0%), systolic pressure (SUCRA: 92.5%), and increased high-density lipoprotein (SUCRA: 100.0%). Aerobic exercise significantly reduced insulin (SUCRA: 89.1%) and HbA1c (SUCRA: 95.3%). Concurrent training significantly reduced HOMA-IR (SUCRA: 93.8%), diastolic blood pressure (SUCRA: 71.2%) and Glucose (SUCRA: 87.6%). Exercise has a significant impact on intermediate disease markers in populations with overweight and obese. Compared with no exercise, exercise lowers total cholesterol, triglycerides, LDL, systolic blood pressure, diastolic blood pressure, HOMA-IR, insulin, and HbA1c, and increases HDL in people with overweight and obese. These findings provide evidence-based recommendations for exercise interventions aimed at weight reduction and the prevention of chronic diseases in individuals with overweight and obese.

## Introduction

The issue of overweight and obesity, characterized by an excessive accumulation of body fat, has emerged as a global health concern in accordance with the World Health Organization (WHO)^1^. Between 1975 and 2016, the worldwide prevalence of obesity tripled, resulting in a scenario where obese individuals outnumber those who are underweight, except for regions such as sub-Saharan Africa and Asia. Obesity is linked to multiple chronic diseases including type 2 diabetes, metabolic syndrome, cardiovascular disease, and certain cancers; this underscores its significant impact on health^2–6^.

Enhanced prevention and management strategies for obesity and its associated chronic diseases have been identified, with exercise playing a pivotal role. Recognizing this significance, the World Health Organization (WHO) has introduced the "Global Action Plan on Physical Activity 2018–2030" to promote physical activity worldwide. Regular exercise not only aids in reducing body fat through increased energy expenditure but also confers benefits such as improved blood lipid profiles, delayed onset of type 2 diabetes, reduced blood pressure, and enhanced mental health^7–10^. Importantly, physical activity is devoid of adverse effects; however, it is advisable to seek professional medical advice before initiating a new exercise regimen, particularly for individuals with pre-existing health conditions^11–13^.

However, the challenge lies in the dearth of evidence-based guidelines for selecting an efficacious exercise regimen that effectively targets specific intermediate disease markers in overweight and obese individuals. The appropriate exercise program has the potential to ameliorate these markers, facilitate weight loss, and mitigate the duration and adverse effects associated with conventional pharmacotherapies, thereby contributing to chronic disease prevention.

This paper employs network meta-analysis, a contemporary evidence-based methodology that assesses the relative efficacy of different treatments^14^, to comprehensively evaluate the impact of multiple exercise interventions on disease markers in overweight and obese individuals. By integrating both direct and indirect evidence from various studies, this study examines the effects of aerobic, resistance, and concurrent training on metabolic markers. The findings provide a solid foundation for developing effective exercise programs for weight management and disease prevention, aiming to inform healthcare professionals, researchers, and policymakers about promoting physical activity..

## Materials and methods

### Search strategy

The researchers in this paper searched five electronic databases (PubMed, EMBASE, Cochrane Central Register of Controlled Trials, Web of Science and CNKI) from January 2017 to March 2024. The search strategy was constructed around the PICOS tool: (P) Population: people with overweight and obese; (I) Intervention: exercise; (C) Comparator: Carry out normal daily activities without calorie restriction according to habitual lifestyle, and do not participate in any physical activities during the experiment; (O) Outcomes: intermediate disease markers in overweight and obese people; (S) Study type: RCTs. The detailed search strategy is shown in Table 1 (PubMed is used as an example).

**Table 1:** Search strategy on PubMed.

	Search strategy
#1	"Overweight and obesity"[MeSH Major Topic]
#2	(((((((((((((((((((((Obesity, Pediatric[Title/Abstract]) OR (Obesity in Childhood[Title/Abstract])) OR (Childhood Onset Obesity[Title/Abstract])) OR (Obesity, Childhood Onset[Title/Abstract])) OR (Child Obesity[Title/Abstract])) OR (Obesity, Child[Title/Abstract])) OR (Childhood Obesity[Title/Abstract])) OR (Obesity, Childhood[Title/Abstract])) OR (Adolescent Overweight[Title/Abstract])) OR (Overweight, Adolescent[Title/Abstract])) OR (Infant Overweight[Title/Abstract])) OR (Overweight, Infant[Title/Abstract])) OR (Adolescent Obesity[Title/Abstract])) OR (Obesity, Adolescent[Title/Abstract])) OR (Obesity in Adolescence[Title/Abstract])) OR (Childhood Overweight[Title/Abstract])) OR (Overweight, Childhood[Title/Abstract])) OR (Infantile Obesity[Title/Abstract])) OR (Obesity, Infantile[Title/Abstract])) OR (Infant Obesity[Title/Abstract])) OR (Obesity, Infant[Title/Abstract])) OR (((((Adult[Title/Abstract]) OR (Overweight, Adult[Title/Abstract])) OR (Obesity, Adult[Title/Abstract])) OR (Adult Obesity[Title/Abstract])) OR (Obesity in Adults[Title/Abstract])) OR (Overweight in Adults[Title/Abstract])) OR (Adult Onset Obesity[Title/Abstract])) OR (Obesity, Adult Onset[Title/Abstract])) OR (Adult Overweight[Title/Abstract])) OR (Overweight, Adult[Title/Abstract])) OR (Adulthood Obesity[Title/Abstract])) OR (Obesity, Adulthood[Title/Abstract])) OR (Adulthood Overweight[Title/Abstract])) OR (Overweight, Adulthood[Title/Abstract]))
#3	#1 OR #2
#4	"exercise"[MeSH Major Topic]
#5	(((((((((((((((((((((((((Physical Activity[Title/Abstract])) OR (Activities, Physical[Title/Abstract])) OR (Activity, Physical[Title/Abstract])) OR (Physical Activities[Title/Abstract])) OR (Exercise, Physical[Title/Abstract])) OR (Exercises, Physical[Title/Abstract])) OR (Physical Exercise[Title/Abstract])) OR (Physical Exercises[Title/Abstract])) OR (Acute Exercise[Title/Abstract])) OR (Acute Exercises[Title/Abstract])) OR (Exercise, Acute[Title/Abstract])) OR (Exercises, Acute[Title/Abstract])) OR (Exercise, Isometric[Title/Abstract])) OR (Exercises, Isometric[Title/Abstract])) OR (Isometric Exercises[Title/Abstract])) OR (Isometric Exercise[Title/Abstract])) OR (Exercise, Aerobic[Title/Abstract])) OR (Aerobic Exercise[Title/Abstract])) OR (Aerobic Exercises[Title/Abstract])) OR (Exercises, Aerobic[Title/Abstract])) OR (Exercise Training[Title/Abstract])) OR (Exercise Trainings[Title/Abstract])) OR (Training, Exercise[Title/Abstract])) OR (Trainings, Exercise[Title/Abstract])
#6	#4 OR #5
#7	"Cholesterol, LDL"[MeSH Major Topic]
#8	(((((((((((((((((((((((((((((((((((((((((((((((((((((((((((((((((((((((((((((((((Low Density Lipoprotein Cholesterol[Title/Abstract])) OR (beta-Lipoprotein Cholesterol[Title/Abstract]))) OR (Cholesterol, beta- Lipoprotein[Title/Abstract])) OR (beta Lipoprotein Cholesterol[Title/Abstract])) OR (LDL Cholesterol[Title/Abstract])) OR (Cholesteryl Linoleate, LDL[Title/Abstract])) OR (LDL Cholesteryl Linoleate[Title/Abstract])) OR (Triglycerides[MeSH Major Topic])) OR (Triacylglycerols[Title/Abstract])) OR (Triacylglycerol[Title/Abstract])) OR (Triglyceride[Title/Abstract])) OR (Glycated Hemoglobin A[MeSH Major Topic])) OR (Hemoglobin A, Glycated[Title/Abstract])) OR (Hb A1a + b[Title/Abstract])) OR (Hb A1c[Title/Abstract])) OR (HbA1[Title/Abstract])) OR (Glycosylated Hemoglobin A[Title/Abstract])) OR (Hemoglobin A, Glycosylated[Title/Abstract])) OR (Hb A1[Title/Abstract])) OR (Glycohemoglobin A[Title/Abstract])) OR (Hemoglobin A(1[Title/Abstract]))) OR (Hemoglobin, Glycosylated A1a-1[Title/Abstract])) OR (A1a-1 Hemoglobin, Glycosylated[Title/Abstract])) OR (Glycosylated A1a-1 Hemoglobin[Title/Abstract])) OR (Hemoglobin, Glycosylated A1a 1[Title/Abstract])) OR (Hb A1a-1[Title/Abstract])) OR (Hb A1a- 2[Title/Abstract])) OR (Hemoglobin, Glycated A1a-2[Title/Abstract])) OR (A1a-2 Hemoglobin, Glycated[Title/Abstract])) OR (Glycated A1a-2 Hemoglobin[Title/Abstract])) OR (Hemoglobin, Glycated A1a 2[Title/Abstract])) OR (Glycated Hemoglobin A1c[Title/Abstract])) OR (Hemoglobin A1c, Glycated[Title/Abstract])) OR (Glycosylated Hemoglobin A1c[Title/Abstract])) OR (Hemoglobin A1c, Glycosylated[Title/Abstract])) OR (Glycated Hemoglobins[Title/Abstract])) OR (Hemoglobins, Glycated[Title/Abstract])) OR (Hemoglobin, Glycosylated[Title/Abstract])) OR (Glycosylated Hemoglobin[Title/Abstract])) OR (Glycated Hemoglobin[Title/Abstract])) OR (Hemoglobin, Glycated[Title/Abstract])) OR (Hemoglobin, Glycated A1b[Title/Abstract])) OR (Hemoglobin, Glycated A1b[Title/Abstract])) OR (A1b Hemoglobin, Glycated[Title/Abstract])) OR (Glycated A1b Hemoglobin[Title/Abstract])) OR (Hb A1b[Title/Abstract])) OR (Hemoglobin, GlycosylatedA1b[Title/Abstract])) OR (A1b Hemoglobin, Glycosylated[Title/Abstract])) OR (Glycosylated A1b Hemoglobin[Title/Abstract])) OR (Cholesterol, HDL[MeSH Major Topic])) OR (alpha-Lipoprotein Cholesterol[Title/Abstract])) OR (Cholesterol, alpha-Lipoprotein[Title/Abstract])) OR (alpha Lipoprotein Cholesterol[Title/Abstract])) OR (HDL Cholesterol[Title/Abstract])) OR (High Density Lipoprotein ^Cholesterol[Title/Abstract])) OR (Cholesterol, HDL2[Title/Abstract]))^ OR (HDL2 Cholesterol[Title/Abstract])) OR (HDL(2) ^Cholesterol[Title/Abstract])) OR (Cholesterol, HDL3[Title/Abstract]))^ OR (HDL3 Cholesterol[Title/Abstract])) OR (HDL(3) Cholesterol[Title/Abstract])) OR (Blood Pressure[MeSH Major Topic])) OR (Pressure, Blood[Title/Abstract])) OR (Diastolic Pressure[Title/Abstract])) OR (Pressure, Diastolic[Title/Abstract])) OR (Pulse Pressure[Title/Abstract])) OR (Pressure, Pulse[Title/Abstract])) OR (Systolic Pressure[Title/Abstract])) OR (Pressure, Systolic[Title/Abstract])) OR (Pressures, Systolic[Title/Abstract])) OR (C-Reactive Protein[MeSH Major Topic])) OR (C Reactive Protein[Title/Abstract])) OR (hsCRP[Title/Abstract])) OR (High Sensitivity C-Reactive Protein[Title/Abstract])) OR (High Sensitivity C Reactive Protein[Title/Abstract])) OR (hs-CRP[Title/Abstract])) OR (homeostasis model assessment of insulin resistance[Title/Abstract])) OR (total cholesterol[Title/Abstract])) OR (fasting glucose[Title/Abstract])) OR (intermediate disease markers[Title/Abstract])
#9	#7 OR #8
#10	#3 AND #6 AND #9

### Inclusion criteria

(1) Participants were only overweight (BMI ≥ 25 kg/m^2^) or obese (BMI ≥ 30 kg/m^2^) and had no other medical conditions; (2) The experimental group administered exercise interventions only to overweight and obese people; (3) Ability to determine the isolated effect of movement; (4) People in the control group did not participate in any intervention and only performed normal daily activities; (5) Clinical randomized controlled trial; (6) Outcome indicators including at least one of the following: Total Cholesterol, Triglycerides, High-density lipoprotein, Low-density lipoprotein, Systolic Blood Pressure, Diastolic Blood Pressure, Homeostatic Model Assessment of Insulin Resistance, Glucose, Insulin, Hemoglobin A1c.

### Exclusion criteria

(1) Studies with incomplete or unreported data (2) Studies from non-randomized controlled trials [including quasi-randomized controlled trials, protocols, conference abstracts, case reports or correspondence] (3) Research on animal experiments.

### Study selection

The screening and exclusion of literature was conducted using the EndNote literature management software. The process began with two researchers reviewing the titles of the literature to eliminate duplicates and any non-randomized controlled trial studies, review papers, conference papers, protocols, and correspondence. Next, both researchers read the abstracts of the remaining literature to determine which studies to include and which to exclude. Finally, the full text of the remaining studies was read by both researchers to confirm their inclusion. If there were any discrepancies in the final selection of studies, the two researchers discussed and resolved the issue with the assistance of a third researcher.

### Data extraction

A seven-item, standardized and pre-selected data extraction form was used to record data for inclusion in the study under the following headings: (1) author, (2) year of publication, (3) country, (4) study period, (5) sample size, (6) mean age, and (7) details of the exercise intervention.

### Risk of bias evaluation of the included RCTs

According to the Cochrane Intervention Systematic Review Manual, two review authors assessed the risk of literature bias using the widely recognized Cochrane tool, considered the gold standard for assessing risk of bias in randomized controlled trials (RCTs).The assessment was based on six key components: selection bias, including the proper generation of random sequences and allocation concealment; performance bias, including participant and personnel blinding; detection bias, including outcome assessment blinding; attrition bias, which addresses incomplete outcome data; reporting bias, which examines selective reporting; and other bias, such as crossover carryover effects in RCTs^15^. The results of this assessment were clearly and concisely presented visually. We also used the STATA software to generate funnels to assess potential publication bias in small studies based on symmetry criteria.

### Data analysis

In this study, we conducted a meta-analysis to investigate the effects of various exercise interventions on disease markers among overweight and obese people. To maintain consistency in the studies, all interventions were quantified as continuous variables. We used a random-effects model to accommodate any differences between studies. Subsequent analysis was carried out using the STATA 15.1 software. Here, we used standardized mean differences (SMDs) to measure different effect sizes between different interventions. In addition, 95% confidence intervals were used to estimate the possible range of these differences.

We plan to employ the nodal method as a means of quantifying and demonstrating the concordance between indirect and direct comparisons.

These calculations will be conducted using the Stata software. If the P-value is greater than 0.05, it would indicate a successful consistency test^16^. To visualize the relationships between different motor interventions, a network diagram was utilized. This diagram, generated using Stata software, depicted each mode of intervention as a node, with the connecting lines between nodes representing direct comparisons. The size of each node reflected the volume of literature related to the corresponding exercise intervention, and the thickness of connecting lines represented the number of studies comparing the interventions^17^.

The Cumulative Ranking Curve (SUCRA) method was used to rank the relative effectiveness of each intervention. This method converts the therapeutic effect of each intervention into an area chart, where the values of the area and area under the curve represent the likelihood of the optimal intervention. In this study, the larger the area under the curve and the closer the value was to 100%, the better the effect of the intervention. In this study, the larger the area under the curve, the closer the value was to 100%, the better the effect of the intervention.

## Results

### Study and identification and selection

**Figure 1:**
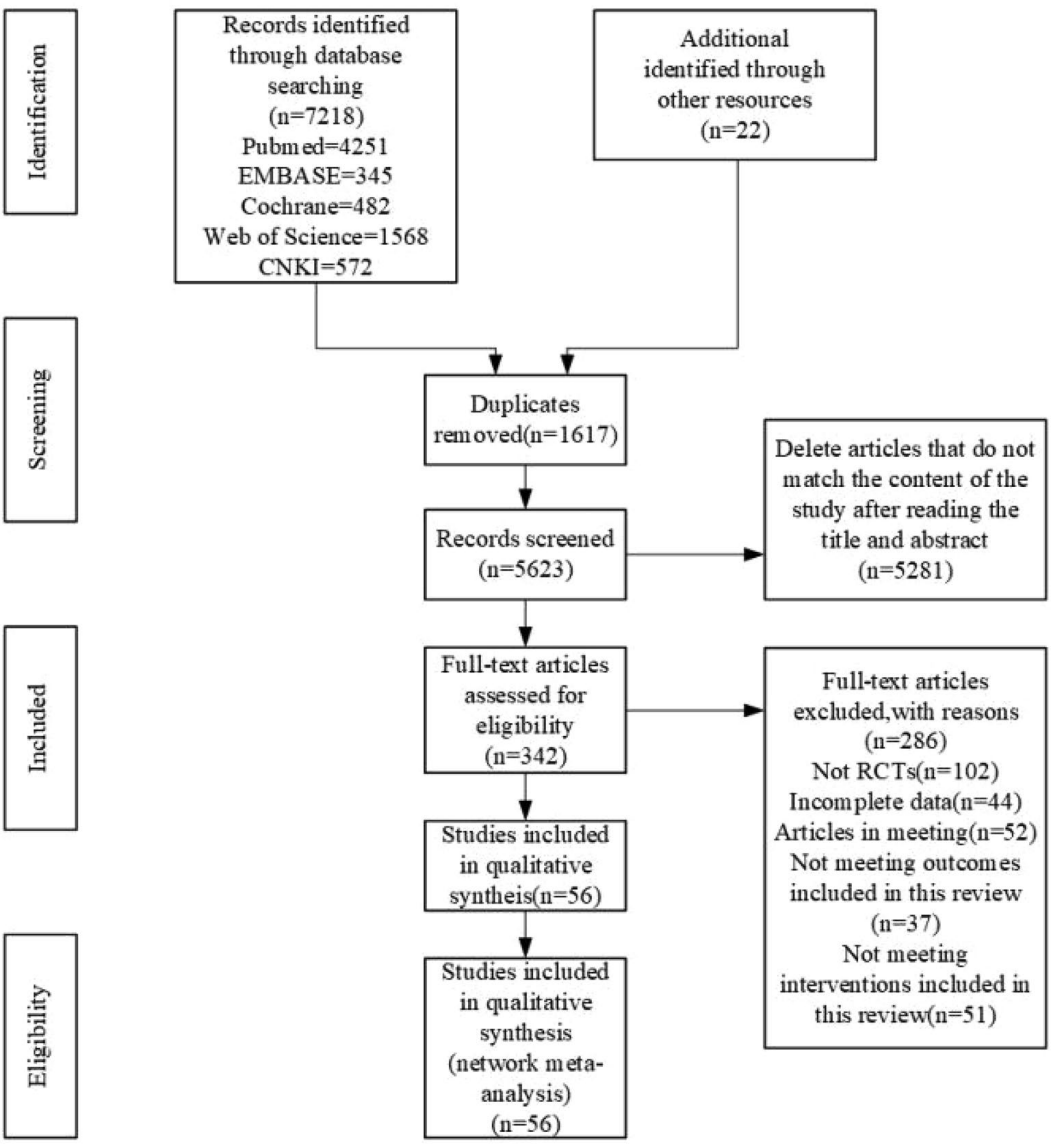
Flow diagram of literature selection.

A total of 7218 documents were retrieved from the electronic database, and an additional 22 documents were manually searched. After eliminating duplicates, the remaining 5623 documents were read for titles and abstracts, and 5281 articles were excluded due to irrelevance after reviewing their titles and abstracts. The remaining 342 documents were read in full and 286 documents were again excluded (for reasons including: non-randomized controlled trials, incomplete data, conference papers and failure to meet the interventions included in this review), leaving a final remaining 56 documents to be included in this study (Fig. 1).

### Quality assessment of the included studies

**Figure 2:**
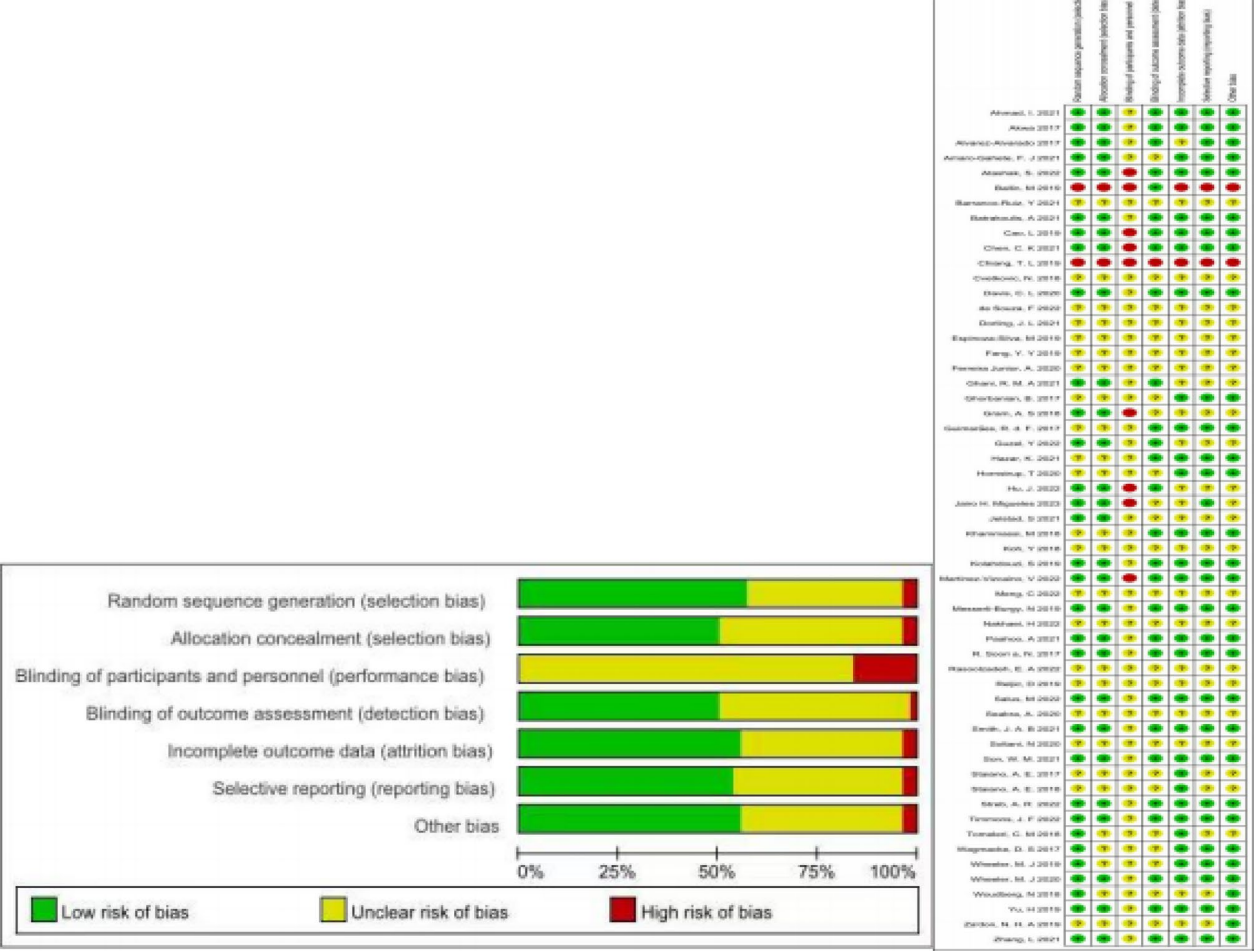
Risk of bias summary & risk of bias graph.

Forty-seven subjects were classified as medium risk, and nine as high risk. We found that no study achieved complete blinding of both subjects and measurers. Specific details will be presented in Fig. 2.

### Characteristics of the included studies

In total, we included studies from 56 randomized controlled trials, which included 3193 people diagnosed with overweight and obesity. Interventions in the experimental group included: aerobic exercises (32 studies)^18–34^, resistance training (9 studies)^35–43^, concurrent training (23 studies)^23,29,34,44–54^.

Forty-seven studies reported TC as an outcome indicator,49 studies reported TG as an outcome indicator, 48 studies reported HDL as an outcome indicator, 48 studies reported LDL as an outcome indicator, 37 studies reported SBP as an outcome indicator, 35 studies reported DBP as an outcome indicator, 34 studies reported Glu as an outcome indicator, 20 studies reported insulin as an outcome indicator, 23 studies reported HOMA-IR as an outcome indicator and 11 studies reported Hba1c as an outcome indicator.

There were six studies from USA, six studies from Brazil, one study from South Africa, seven studies from Iran, seven studies from China, four studies from the Spain, one study from Saudi Arabia, one study from Ghana, one study from The Republic of Azerbaijan, one study from Serbia, two studies from Denmark, one study from Tunisia, one study from Beaumont, two studies from Norway, one study from Chile, one study from Switzerland, one study from Germany, two studies from Australia, one study from Portugal, one study from Saudi Arabia, one study from Greece, two studies from Malaysia, one study from Egypt, one study from Sweden, one study from Korea, one study from Turkey, one study from Estonia, one study from Ireland. The characteristics of the included studies are shown in Table 2.

**Table 2:** Characteristics of the studies included in the meta-analysis.

Author	Country	Year	Population	Age (mean + SD)	Total/male/female	Intervention	Control	Outcome
Akwa	Ghana	2017	Overweight and obesity	T: 60.99 (11.88) C: 59.91 (9.03)	T: 8/NA/8 C: 10/NA/10	Aerobic exercise Length of Intervention:8 weeks Freq:3 times a week Duration:60 min	CON	SBP, DBP, LDL, HDL, TG, TC
Alvarez-Alvarado	USA	2017	Overweight and obesity	T: 20 (5) C: 21 (3.6)	T: 20/NA/20 C: 21/NA/21	RT (whole-body vibration training) Length of Intervention:6 weeks Freq:3 times a week Duration:11–30 min	CON	SBP, DBP
Ghorbanian, B	The Republic of Azerbaijan	2017	Overweight and obesity	T: 21.1 (2.46) C: 22.2 (1.88)	T: 10/NA/10 C: 10/NA/10	RT (rope training) Length of Intervention:8 weeks Freq:4times a week Duration:45 min	CON	TC, TG, HDL, LDL, Glu, HOMA-IR, insulin
Staiano, A. E	USA	2017	Overweight and obesity	T: 15.3 (1.2) C: 16.1 (1.4)	T: 20/NA/20 C: 18/NA/18	Aerobic exercise (dance) Length of Intervention:12 weeks Freq:3 times a week Duration:60 min	CON	SBP, DBP, TC, TG, HDL, LDL, Glu, insulin
Wagmacke, D. S	Brazil	2017	Overweight and obesity	T + C: 24 (3.6)	T: 33/NA/33 C: 33/NA/33	Aerobic exercise (running) Length of Intervention: 1 week Freq: 1 time a week Duration:12 min	CON (sedentary)	HOMA-IR,TC,TG, HDL,LDL, insulin, Glu
Guimarães, R. d. F	Brazil	2017	Overweight and obesity	T: 16.0 (0.9) C: 17.1 (0.7)	T: 26/NA/NA C: 45/NA/NA	Aerobic exercise Length of Intervention:14 weeks Freq: 2 times a week Duration: 60 min	CON	TC, TG, SBP, DBP
R. Soori a, N	Iran	2017	Obesity	T + C: NA	T: 24/NA/24 C: 8/NA/8	RT (water based)/Aerobic exercise (water based)/CT (water based) Length of Intervention: 10 weeks Freq: 3 times a week Duration: 45 min	CON	TC,TG, HDL, LDL, HOMA-IR
Cvetkovic, N	Serbia	2018	Overweight and obesity	T + C: NA	T: 21/NA/NA C: 14/NA/NA	Aerobic exercise(football)/CT(HIIT) Length of Intervention: 12 weeks Freq: 3 times a week Duration: 60 min	CON	SBP, DBP
Gram, A. S	Denmark	2018	Overweight and obesity	T: 35 (7.78) T: 33 (7.10) T: 36 (7.50) C: 35 (8.16)	T: 74/37/37 C: 16/9/7	Aerobic exercise (bike)/CT/CT Length of Intervention: 6 months Freq: 5 times a weeks Duration: 60 min	CON	TC, TG, HDL, LDL
Khammassi, M	Tunisia	2018	Overweight and obesity	T + C: 19.4 (1.1)	T: 10/NA/NA C: 10/NA/NA	CT(HIIT) Length of Intervention: 12 weeks Freq: 3 times a week Duration: 15–27 min	CON	TC, TG, HDL, LDL
Koh, Y	Beaumont	2018	Obesity	T: 30.00 (16.00) C: 25.00 (8.00)	T: 15/8/7 C: 12/6/6	Aerobic exercise (running) Length of Intervention: 4 weeks Freq: 3 nonconsecutive days per week Duration: 60 min	CON	TC, TG, HDL, LDL
Staiano, A. E	USA	2018	Overweight and obesity	T + C: 11 (1)	T: 22/NA/NA C: 23/NA/NA	Aerobic exercise Length of Intervention: 24 weeks Freq: 3 times a week Duration: 60 min	CON	SBP, DBP, TC, TG, HDL, LDL, Glu
Tomeleri, C. M	Brazil	2018	Overweight and obesity	T + C: 70.4 (5.7)	T: 26/NA/26 C: 27/NA/27	RT Length of Intervention: 12 weeks Freq: 3 times a week Duration: 30 min	CON	HOMA-IR, TG, HDL, SBP, Glu, DBP
Woudberg, N	South Africa	2018	Obesity	T: 22.8 (0.7) C: 24.5 (0.9)	T: 20/NA/20 C: 15/NA/15	CT/RT Length of Intervention: 12 weeks Freq: 4 times a week Duration: 40–60 min	CON	TC, LDL, HDL
Ballin, M	Norway	2019	Obesity	T + C: 70 (NA)	T: 36/NA/NA C: 36/NA/NA	CT (vigorous interval training) Length of Intervention: 10 weeks Freq: 3 times a week Duration: 33–51 min	CON	SBP, DBP, TC, TG, HDL, LDL
Cao, L	China	2019	Overweight and obesity	T: 63.8 (5.9) C: 64.0 (4.6)	T: 13/NA/NA C: 15/NA/NA	Aerobic exercise Length of Intervention: 12 weeks Freq: 3 times a week Duration: 20–40 min	CON	SBP, DBP, TC, TG, HDL, LDL
Chiang, T. L	China (Taiwan)	2019	Obesity	T + C: 19.7 (0.80)	T: 23/NA/NA C: 9/NA/NA	Aerobic exercise (walk) Length of Intervention: 8 weeks Freq: 5 times a week Duration: 30 min	CON	SBP, DBP, HDL, Glu, TG
Espinoza-Silva, M	Chile	2019	Overweight and obesity	T + C(male): 8.29 (1.53) T + C (female): 7.99 (1.47)	T: 69/NA/NA C: 30/NA/NA	CT (HIIT) Length of Intervention: 28 weeks Freq: 5 times a week Duration: 40–50 min	CON	SBP, DBP
Fang, Y. Y	China (Taiwan)	2019	Overweight and obesity	T + C: NA	T: 37/NA/NA C: 38/NA/NA	Aerobic exercise Length of Intervention: 12 weeks Freq: 3 times a week Duration: 60 min	CON	SBP, DBP, TC, TG, HDL, LDL, Glu
Kolahdouzi, S	Iran	2019	Overweight and obesity	T + C: 23 (3.2)	T: 13/NA/NA C: 13/NA/NA	RT Length of Intervention: 8 weeks Freq: 3 times a week Duration:60 min	CON	TC,TG, HDL, LDL, HOMA-IR, Glu, insulin
Messerli-Burgy, N	Switzerland	2019	Overweight and obesity	T: 8.9 (1.2) C: 9.6 (1.4)	T: 25/NA/NA C: 25/NA/NA	Aerobic exercise Length of Intervention: 1 week Freq: 3 times a week Duration: 30 min	CON (sedentary)	SBP, DBP
Reljic, D	Germany	2019	Overweight and obesity	T + C: 49.8 (13.6)	T: 54/NA/NA C: 33/NA/NA	CT (HIIT/MIIT) Length of Intervention: 12 weeks Freq: 2 times a week Duration: 14 min	CON	TG, SBP, DBP, HDL, Glu, TC, TG, LDL, HbA1c
Wheeler, M. J	Australia	2019	Overweight and obesity	T + C: 67 (7)	T: 134/NA/NA C: 67/NA/NA	Aerobic exercise (walk) Length of Intervention: 1 week Freq: 1 time a week Duration: 30 min	CON (sedentary)	DBP, SBP
Yu, H	China	2019	Overweight and obesity	T: 45.7 (4.0) C: 47.8 (4.8)	T: 13/13/NA C: 10/10/NA	CT (HICT) Length of Intervention: 12 weeks Freq: 6 times a week Duration: 60 min	CON	TC, TG, HDL, LDL, Glu, SBP, DBP
Za'don, N. H. A	Malaysia	2019	Overweight and obesity	T: 29.6 (5.4) C: 29.8 (5.1)	T: 25/11/14 C: 25/14/11	CT (HIIT) Length of Intervention: 12 weeks Freq: 3 times a week Duration: 60 min	CON	Insulin, HOMA-IR
Hornstrup, T	Denmark	2020	Overweight and obesity	T: 46.3 (2.9) T: 45.3 (4.0) C: 41.8 (4.6)	T: 13/NA/13 T: 10/NA/10 C: 9/NA/9	Aerobic exercise (handball training) Length of Intervention: 16 weeks Freq: 3 times a week Duration: 60 min	CON	SBP, DBP, LDL, HDL, TG, TC, HbA1c
Seabra, A	Portugal	2020	Overweight and obesity	T: 11.1 (1.6) C: 10.7 (1.4)	T: 20/20/NA C: 20/20/NA	Aerobic exercise (Soccer Practice) Length of Intervention: 6 months Freq:2 times a week Duration: 45–90 min	CON	TC, TG, HDL, LDL, Glu, HOMA-IR, insulin, SBP, DBP
Davis, C. L	USA	2020	Overweight and obesity	T: 9.6 (0.73) C: 9.7 (0.94)	T: 90/23/67 C: 85/30/55	Aerobic exercise Length of Intervention:8 months Freq: 5 times a week Duration: 40 min	CON (sedentary)	TC, TG, HDL, LDL, Glu, SBP, DBP, HOMA-IR, insulin
Ferreira Junior, A	Brazil	2020	Overweight and obesity	T + C: 47.8 (8.4)	T:25/NA/25 C: 23/NA/23	Aerobic exercise (walk) Length of Intervention: 12 weeks Freq: 3 times a week Duration: 30 min	CON	HOMA-IR, Glu, insulin
Soltani, N	Iran	2020	Overweight and obesity	T + C: 21.5 (3.5)	T: 15/NA/15 C: 15/NA/15	CT (HIIT) Length of Intervention: 10 weeks Freq: 4 times a week Duration: 30 min	CON (sedentary)	TG, HOMA-IR, HDL, Glu,TC, LDL, insulin
Wheeler, M. J	Australia	2020	Overweight and obesity	T + C: 67 (7)	T: 44/NA/NA C: 23/NA/NA	Aerobic exercise (walk) Length of Intervention: 1 week Freq: 1 time a week Duration: 30 min	CON (sedentary)	TG, Glu, insulin
Ahmad, I	Saudi Arabia	2021	Overweight and obesity	T + C: 34.2 (8.9)	T: 75/NA/NA C: 75/NA/NA	RT Length of Intervention: 8 weeks Freq: 3 times a week Duration: NA	CON	TC, TG, HDL, LDL
Amaro-Gahete, F. J	Spain	2021	Overweight and obesity	T: 41.3 (4.4) C: 43.7 (6.1)	T: 6/NA/NA C: 6/NA/NA	CT Length of Intervention: 12 weeks Freq: 3 times a week Duration: 60 min	CON	HOMA-IR,TC,TG, HDL, LDL, SBP, DBP, Glu
Barranco-Ruiz, Y	Spain	2021	Overweight and obesity	T + C: 39.7 (7.4)	T: 25/NA/25 C: 15/NA/15	Aerobic exercise (dance) Length of Intervention: 16 weeks Freq: 3 times a week Duration: 60 min	CON	TC, TG, HDL, LDL, Glu, SBP, DBP
Batrakoulis, A	Greece	2021	Overweight and obesity	T + C: 36.4 (4.4)	T: 28/NA/28 C: 21/NA/21	CT Length of Intervention: 20 weeks Freq: 3 times a week Duration: 6–15 min	CON	TC, TG, HDL, LDL, Glu, SBP, HOMA-IR, DBP, insulin
Chen, C. K	Malaysia	2021	Overweight and obesity	T + C: 23.2 (3.9)	T: 19/NA/19 C: 19/NA/19	Aerobic exercise (walk) Length of Intervention: 6 weeks Freq: 3 times a week Duration:25–35 min	CON	SBP, DBP
Dorling, J. L	USA	2021	Overweight and obesity	T: 46.2 (11.4) C: 46.7 (9.8)	T: 20/3/17 C: 20/6/14	Aerobic exercise Length of Intervention: 3 months Freq: 5 times a week Duration: 127 (61) min	CON (sedentary)	HbA1c, SBP, DBP, TC, TG, HDL, LDL,Glu,HOMA-IR,insulin
Ghani, R. M. A	Egypt	2021	Overweight and obesity	T: 30.86 (3.63) C: 31.10 (3.84)	T: 50/NA/50 C: 50/NA/50	Aerobic exercise (walk) Length of Intervention: 8 weeks Freq: 5 times a week Duration: 15–30 min	CON	HbA1c
Hazar, K	USA	2021	Overweight and obesity	T: 34.95 (9.72) C: 35.27 (8.44)	T: 21/NA/21 C: 20/NA/20	Aerobic exercises (Moderate Intensity Aerobic Exercises) Length of Intervention: 8 weeks Freq: 3 times a week Duration: 60 min	CON	TC, TG, HDL, LDL
Jelstad, S	Norway	2021	Overweight and obesity	T + C:54 (10)	T: 10/10/NA C: 10/10/NA	Aerobic exercise Length of Intervention: 3 weeks Freq: 3 times a week Duration: 60 min	CON	TC, TG, HDL, LDL, Glu, HbA1c, HOMA-IR, insulin
Paahoo, A	Iran	2021	Overweight and obesity	T + C: 11.06 (1.0)	T: 30/30/NA C: 15/15/NA	Aerobic exercises/ CT (HIIT) Length of Intervention: 12 weeks Freq: 3 times a week Duration: 30 min	CON	TC, TG, HDL, LDL
Smith, J. A. B	Sweden	2021	Overweight and obesity	T + C: 48.91 (7.3)	T: 8/NA/NA C: 8/NA/NA	Aerobic exercises Length of Intervention: 3 weeks Freq: everyday Duration: 60 min	Con (sedentary)	HbA1c, TC, TG, HDL, LDL, Glu, HOMA-IR, insulin
Son, W. M	Korea	2021	Overweight and obesity	T: 68.2 (1.6) C: 68.2 (1.4)	T: 18/NA/18 C: 17/NA/17	RT Length of Intervention: 12 weeks Freq: 3 times a week Duration: 60 min	CON	TG, HDL, LDL, Glu, HOMA-IR, insulin, SBP, DBP
Zhang, L	China	2021	Overweight and obesity	T: 19.92 (2.70) C: 20.16 (2.50)	T: 10/10/NA C: 10/10/NA	CT (HIIT) Length of Intervention: 6 weeks Freq: 3 times a week Duration: 30 min	CON	TC, TG, HDL, LDL, Glu, HOMA-IR, insulin, SBP, DBP
Atashak, S	Iran	2022	Overweight and obesity	T: 24.55 (3.21) C: 25.37 (3.01)	T: 15/15/NA C:15/15/NA	CT (HIIT) Length of Intervention: 12 weeks Freq: 3 times a week Duration: 30 min	CON	TC, TG, HDL, LDL, Glu, HOMA-IR,insulin,SBP, DBP
de Souza, F	Brazil	2022	Overweight and obesity	T: 14.32 (1.28) C: 14.50 (1.39)	T: 22/11/11 C: 20/7/13	CT (karate) Length of Intervention: 12 weeks Freq: 3 times a week Duration: 60 min	CON	TC, TG, HDL, LDL, Glu, SBP, DBP
Guzel, Y	Turkey	2022	Overweight and obesity	T: 55.67 (3.44) C: 54.42 (4.01)	T: 12/NA/12 C: 12/NA/12	Aerobic exercises (walk) Length of Intervention: 10 weeks Freq: 3 times a week Duration: 25–40 min	CON	HbA1c, Glu,SBP, HOMA-IR
Hu, J	China	2022	Overweight and obesity	T: 19.20 (1.10) C: 20.20 (0.40)	T: 17/NA/17 C: 13/NA/13	CT (HIIT) Length of Intervention: 4 weeks Freq: 5 times a week Duration: 35 min	CON	TC, TG, HDL, LDL, SBP, DBP
Martinez-Vizcaino, V	Spain	2022	Overweight and obesity	T (male): 9.89 (0.71) T (female): 10.03 (0.69) C (male): 10.12(0.69) C (female): 10.04 (0.72)	T: 248/120/128 C: 239/113/126	CT (HIIT) Length of Intervention: 3 years Freq: 4 times a week Duration: 60 min	CON	HbA1c, SBP, DBP, TC, TG, HDL, LDL, Glu, insulin
Meng, C	China	2022	Overweight and obesity	T(HIIT): 11.4 (0.8) T(MICT): 11.2 (0.7) C: 11.0 (0.7)	T: 23/23/NA C: 13/13/NA	CT (HIIT/MICT) Length of Intervention: 12 weeks Freq: 3 times a week Duration: 11 min/30 min	CON	TC, TG, HDL, LDL, Glu, HOMA-IR, insulin, SBP, DBP
Nakhaei, H	Iran	2022	Overweight and obesity	T (Stationary bicycle): 33.46 (3.52) T (Spinning exercise): 37.2 (3.56) C: 35.86 (3.29)	T: 30/NA/30 C: 15/NA/15	Aerobic exercises (group cycling/stationary bike) Length of Intervention: 6 weeks Freq: 3 times a week Duration: 60 min	CON	TC, TG, HDL, LDL
Rasoolzadeh, E. A	Iran	2022	Overweight and obesity	T(RT):22.4 (1.95) T(ET): 22.62 (2.19) C: 23.12 (1.56)	T: 18/18/NA C: 8/8/NA	RT/ Aerobic exercise Length of Intervention: 8 weeks Freq: 3 times a week Duration: 15–40 min	CON	Glu, HOMA-IR, insulin
Salus, M	Estonia	2022	Overweight and obesity	T: 13.1 (4.86) C: 13.7 (5.99)	T: 14/14/NA C: 14/14/NA	Aerobic exercises (running) Length of Intervention: 12 weeks Freq: 3 times a week Duration: 29–38 min	CON	TC, TG, HDL, LDL, HOMA-IR, insulin, Glu
Streb, A. R	Brazil	2022	Overweight and obesity	T + C: 37 (1)	T: 18/NA/NA C: 18/NA/NA	CT Length of Intervention: 16 weeks Freq: 3 times a week Duration: 60 min	CON	TC, HDL, TG, LDL
Timmons, J. F	Ireland	2022	Overweight and obesity	T: 25.4 (5.2) C: 26.0 (3.6)	T: 9/9/NA C: 9/9/NA	CT Length of Intervention: 8 weeks Freq: 3 times a week Duration: 30 min	CON	TC, TG, HDL, LDL, Glu
Jairo H. Migueles	Spain	2023	Overweight and obesity	T + C: 10 (1.1)	T: 38/NA/NA C: 33/NA/NA	CT (Aerobic exercise: 60 min Resistance exercise: 30 min) Length of Intervention: 20 weeks Freq: 3–5 times a week Duration: 90 min	CON	LDL

*CON* A control group that normally conducts daily life (no exercise), *T* experimental group, *C* control group, *TC* total cholesterol, *TG* triglyceride, *HDL* high-density lipoprotein, *LDL* low density lipoprotein-Cholesterol, *SBP* systolic pressure, *DBP* diastolic blood pressure, *Glu* glucose, *HOMA-IR* homeostatic model assessment for insulin resistance, *HbA1c* hemoglobin A1c, *RT* resistance training, *CT* concurrent training, *HIIT* high-intensity interval training, *MIIT* Moderate-intensity interval training, *MICT* moderate-intensity continuous training, *T + C* The ages of the experimental and control groups were not reported separately in the study, only the overall age was reported, *NA* unavailable, *Freq* frequency.

### Network meta-analysis

The full NMA figure will be shown in Figs. 3A, 4A, 5A, 6A, 7A, 8A, 9A, 10A, 11A, and 12A.

**Figure 3:**
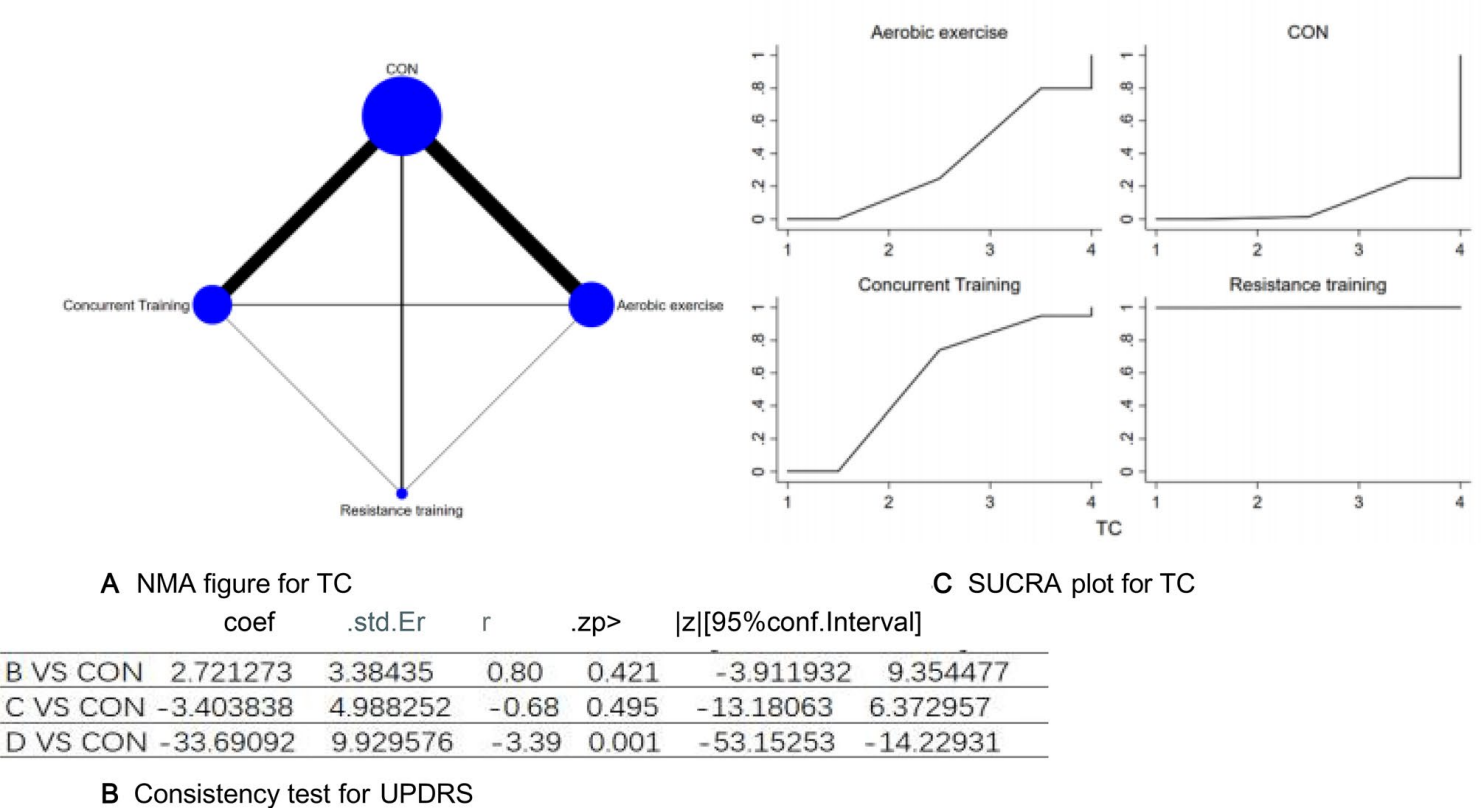
(**A**) NMA figure for TC, (**B**) Consistency test for UPDRS, (**C**) SUCRA plot for TC.

**Figure 4:**
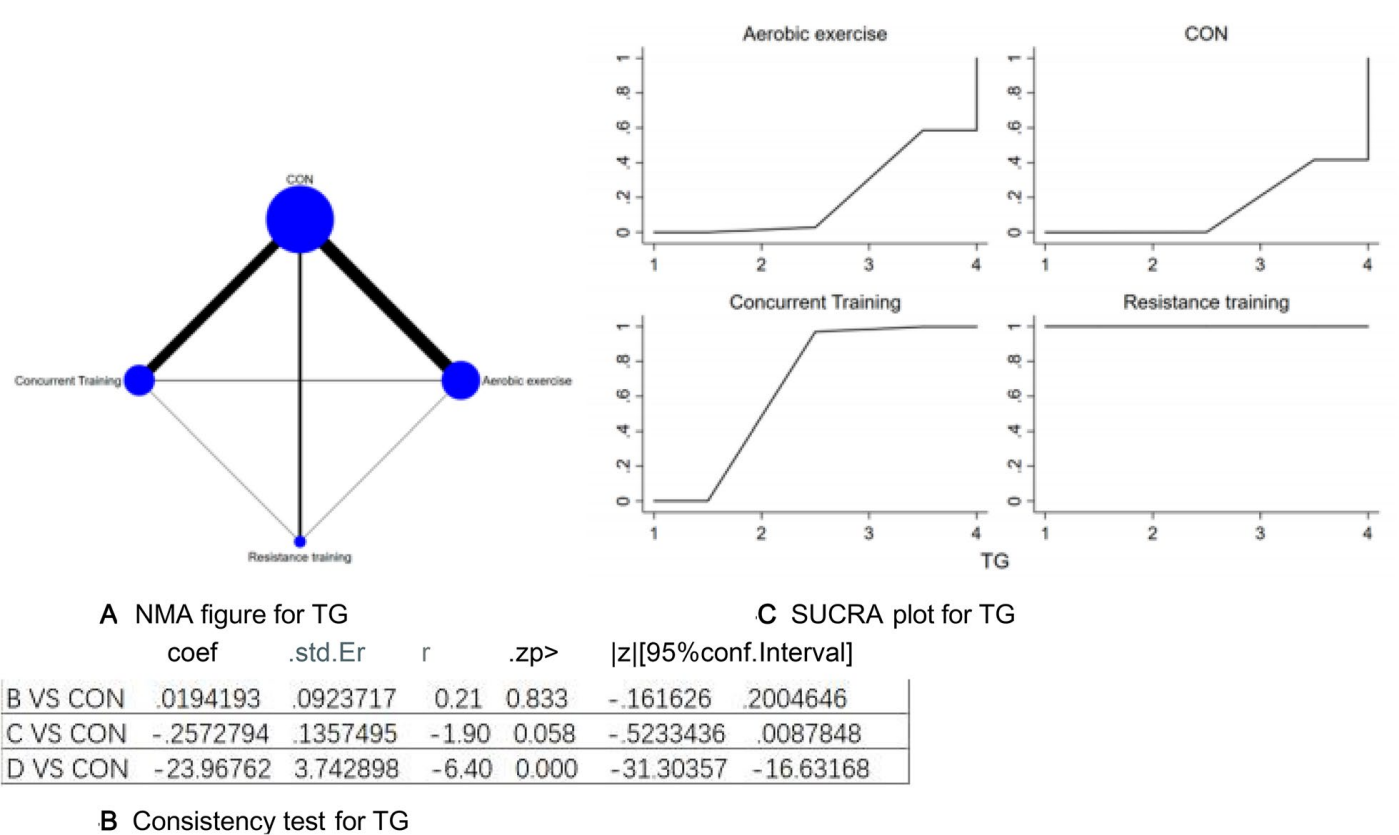
(**A**) NMA figure for TG, (**B**) Consistency test for TG, (**C**) SUCRA plot for TG.

**Figure 5:**
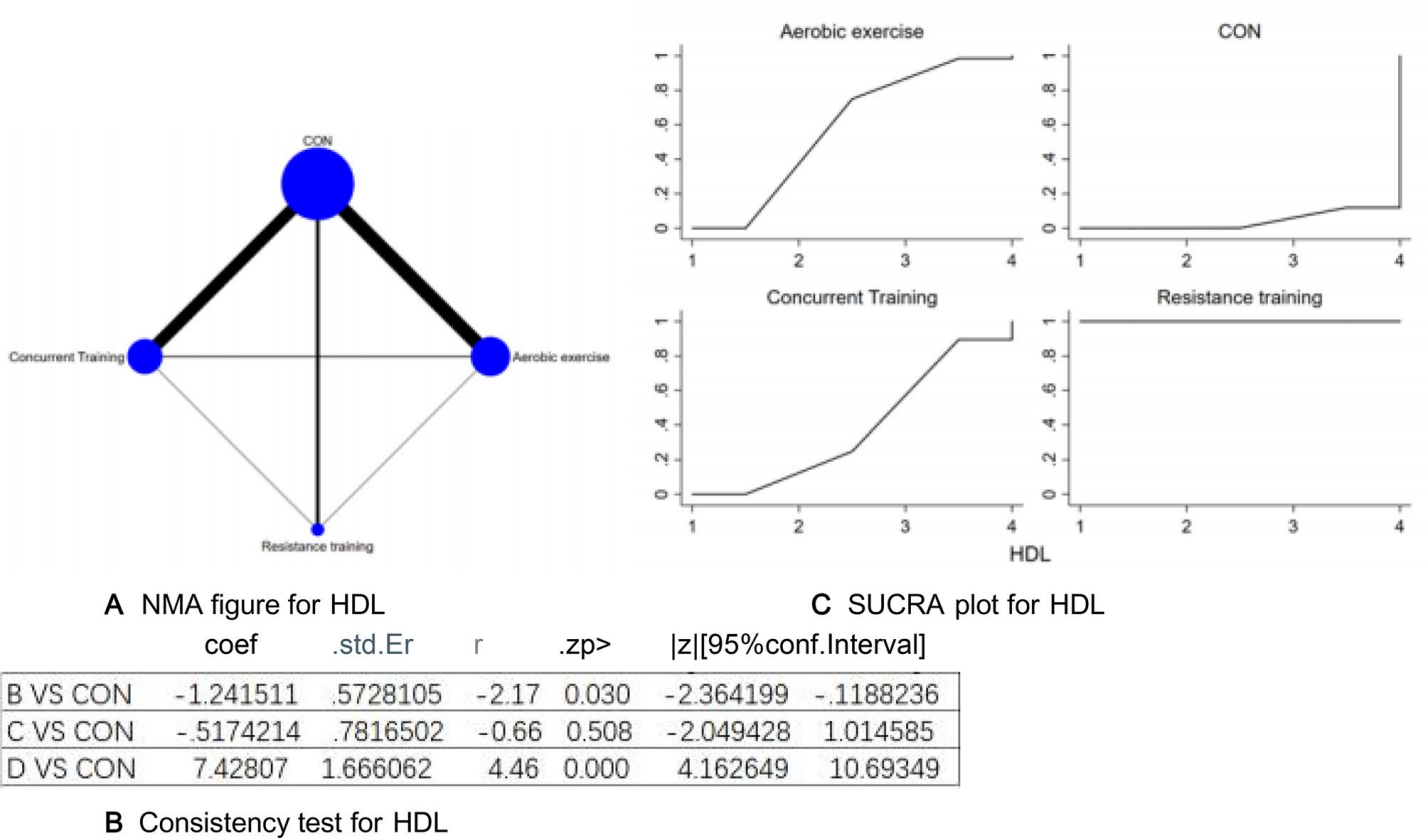
(**A**) NMA figure for HDL, (**B**) Consistency test for HDL, (**C**) SUCRA plot for HDL.

**Figure 6:**
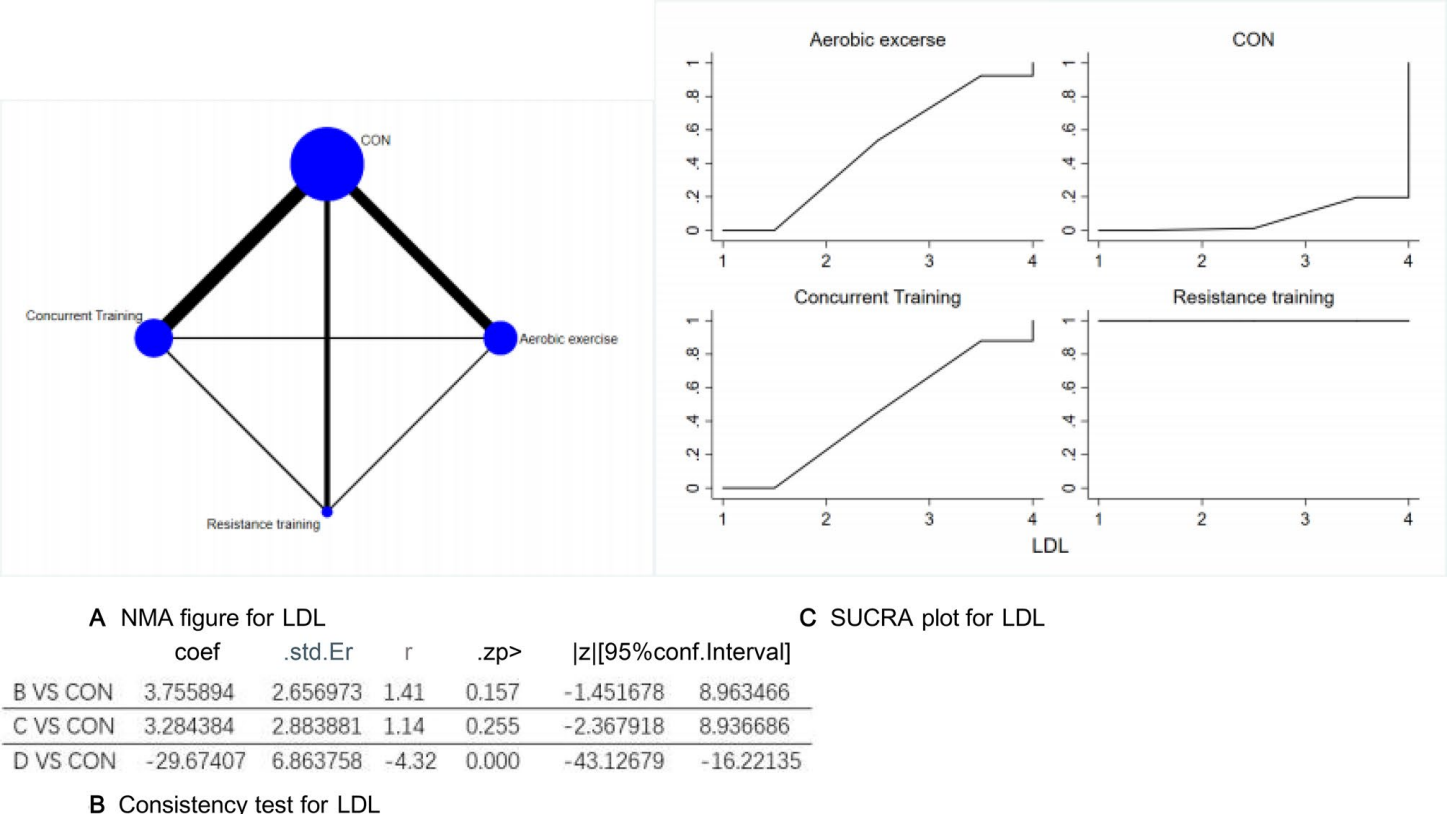
(**A**) NMA figure for LDL, (**B**) Consistency test for LDL, (**C**) SUCRA plot for LDL.

**Figure 7:**
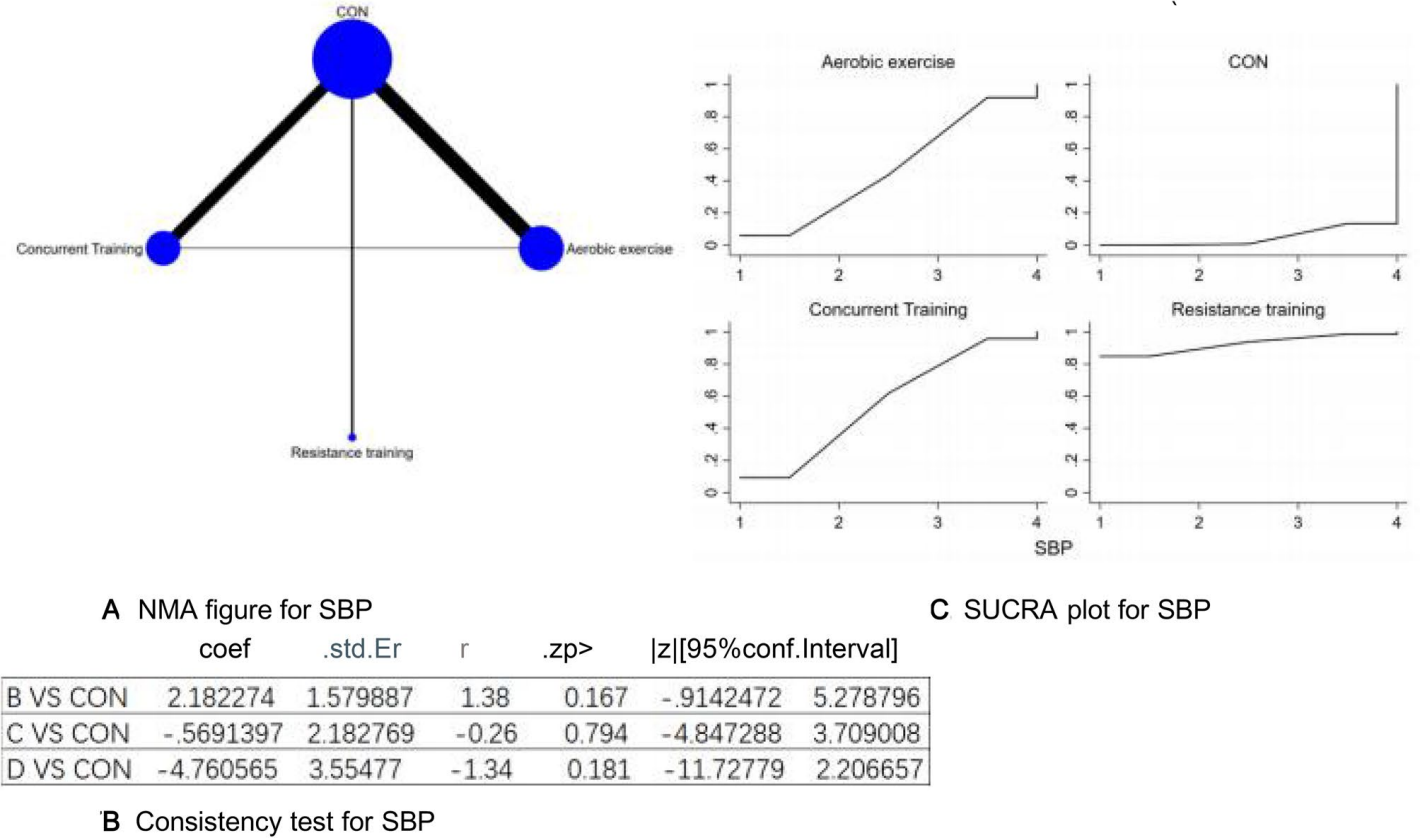
(**A**) NMA figure for SBP, (**B**) Consistency test for SBP, (**C**) SUCRA plot for SBP.

**Figure 8:**
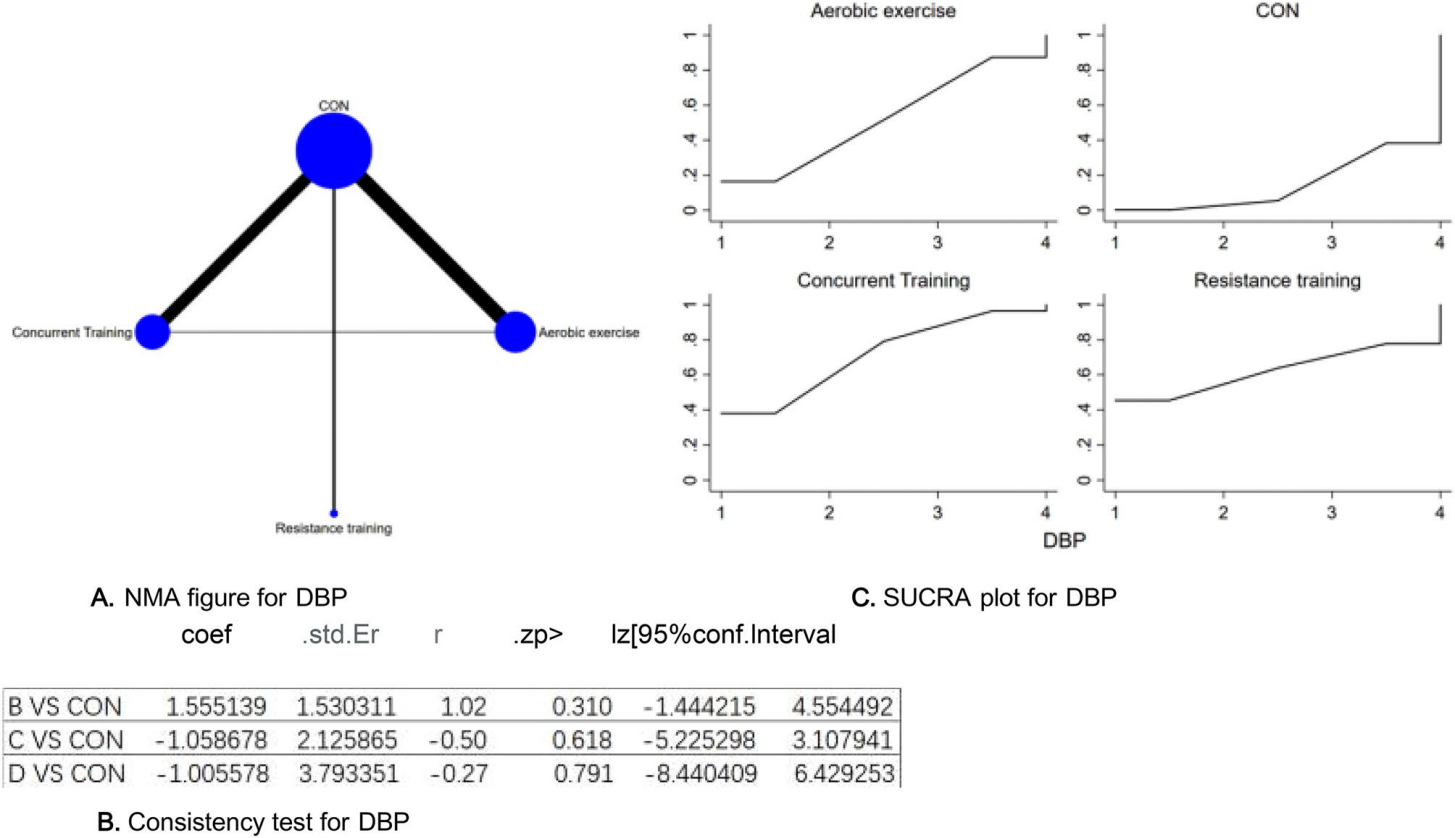
(**A**) NMA figure for DBP, (**B**) Consistency test for DBP, (**C**) SUCRA plot for DBP.

**Figure 9:**
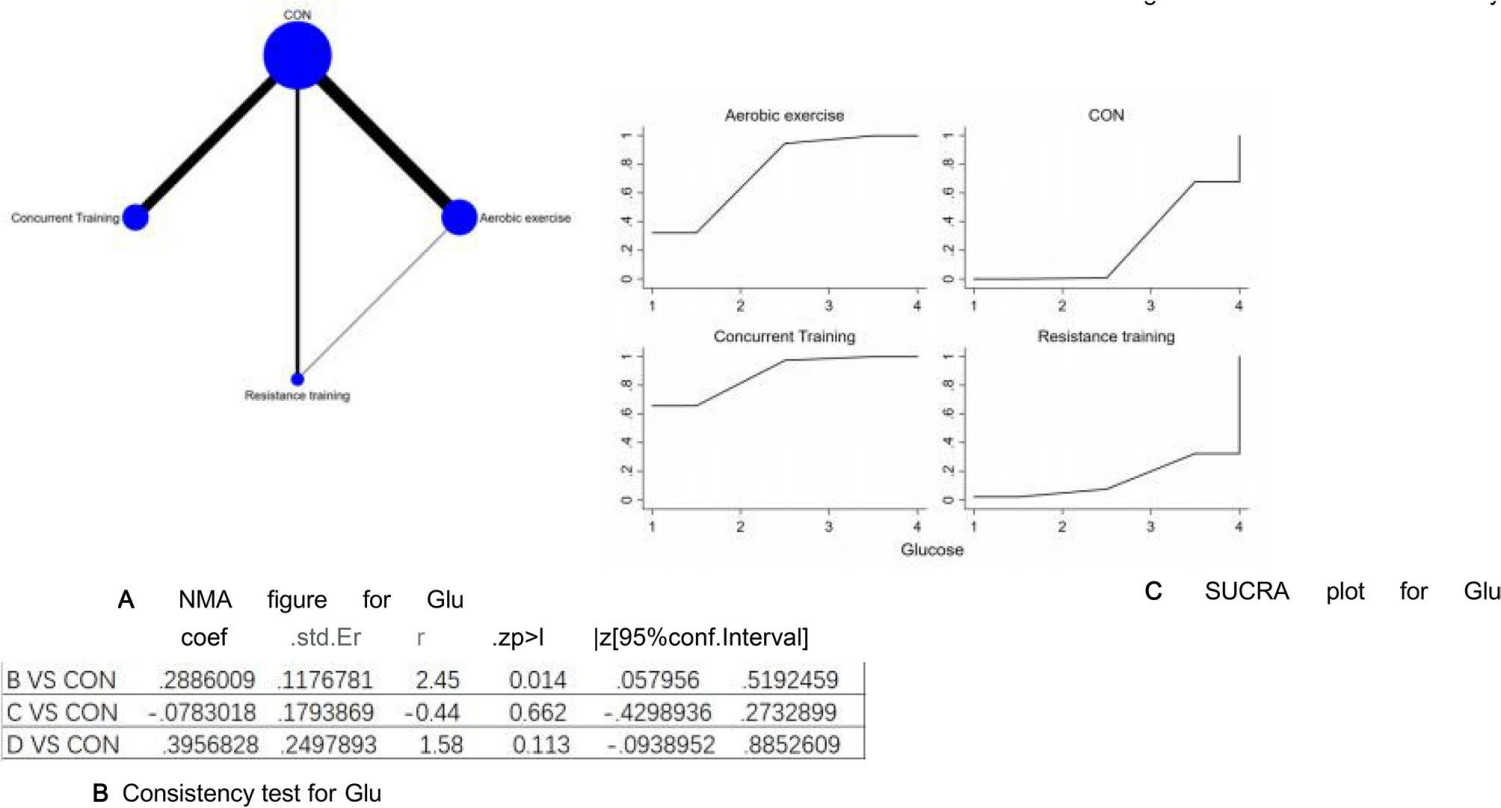
(**A**) NMA figure for Glu, (**B**) Consistency test for Glu, (**C**) SUCRA plot for Glu.

**Figure 10:**
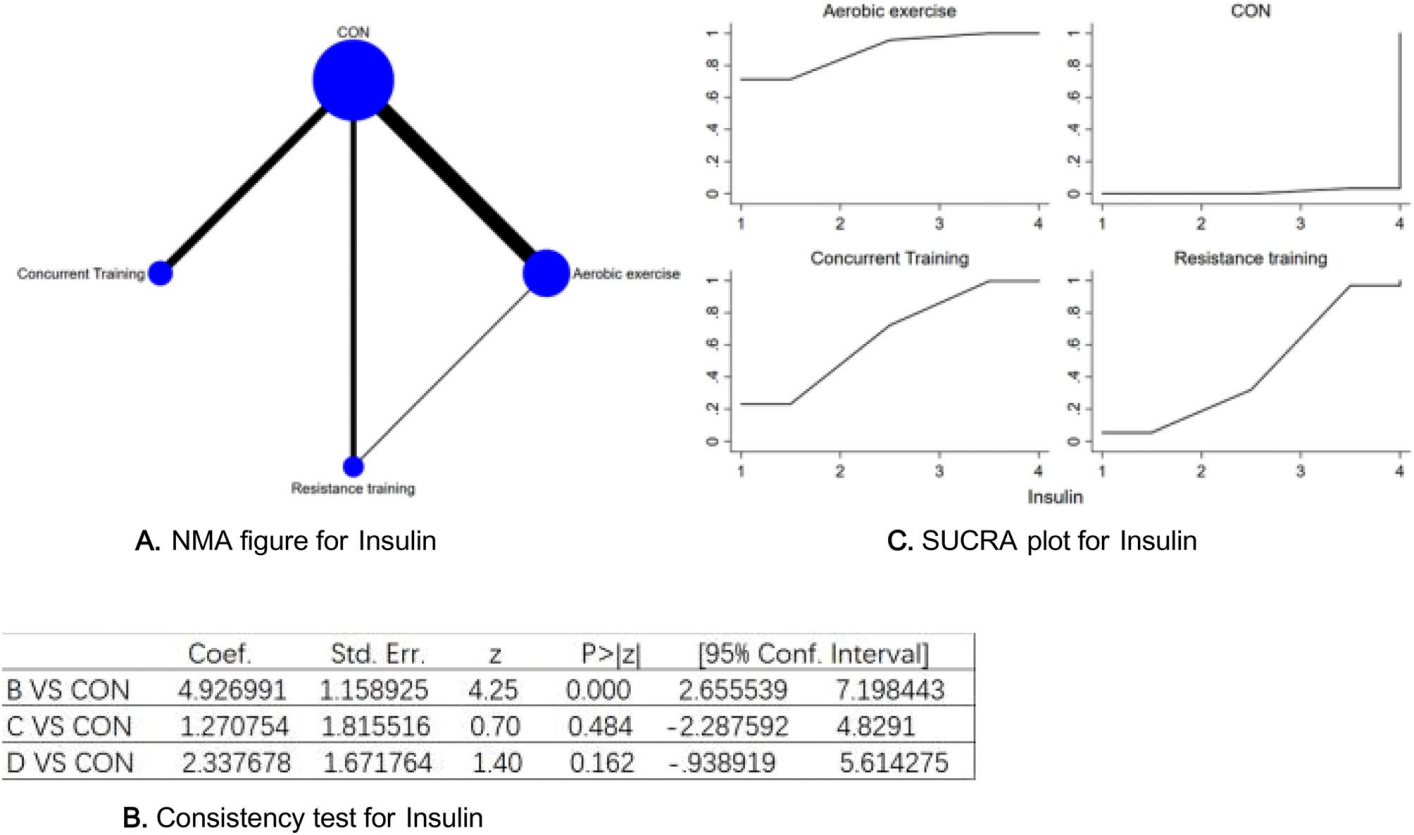
(**A**) NMA figure for Insulin, (**B**) Consistency test for Insulin, (**C**) SUCRA plot for Insulin.

**Figure 11:**
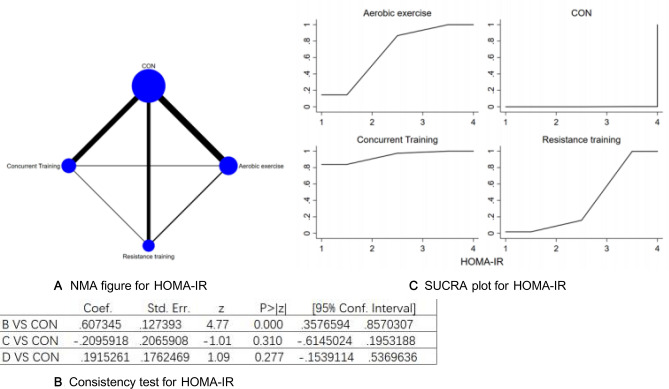
(**A**) NMA figure for HOMA-IR, (**B**) Consistency test for HOMA-IR, (**C**) SUCRA plot for HOMA-IR.

#### Total cholesterol (TC)

All direct and indirect comparisons between all studies were evaluated for consistency and inconsistency, with all P-values exceeding 0.05. This suggests an acceptable level of agreement among the studies. More details will be provided in Fig. 3B.

The results of the Network meta-analysis showed that relative to the control group's routine measures, resistance training [MD = − 30.29, 95% CI (− 50.17, − 10.40)], concurrent training [MD = 30.29, 95% CI (10.40, 50.17)], aerobic exercise [MD = 33.69, 95% CI (14.23, 53.15)]were superior to the control group in reducing TC value, the details will be shown in Table 3. The probability ranking of the different exercise interventions in terms of reducing TC value was ranked first by resistance training in the SUCRA for (SUCRA: 99.9% as shown in Fig. 3C).

**Table 3:** League table on TC.

Aerobic exercise_CON	Concurrent training	Resistance training	Aerobic exercise_CON
Aerobic exercise	2.72 (− 3.91, 9.35)	− 3.40 (− 13.18, 6.37)	− 33.69 (− 53.15, − 14.23)
− 2.72 (− 9.35, 3.91)	CON	− 6.13 (− 13.66, 1.41)	− 36.41 (− 54.86, − 17.97)
3.40 (− 6.37, 13.18)	6.13 (− 1.41, 13.66)	Concurrent training	− 30.29 (− 50.17, − 10.40)
33.69 (14.23, 53.15)	36.41 (17.97, 54.86)	30.29 (10.40, 50.17)	Resistance training

#### Triglycerides (TG)

Each P-value from both direct and indirect comparisons among all studies underwent tests for consistency and inconsistency. Each P-value came out to be above 0.05, which implies a satisfactory degree of concurrence across the studies. Further details will be depicted in Fig. 4B.

The results of the Network meta-analysis showed that relative to the control group's routine measures, resistance training [MD = − 23.71, 95% CI (− 31.05, − 16.37)], concurrent training [MD = 23.71, 95% CI (16.37, 31.05)], aerobic exercise [MD = 23.97, 95% CI (16.63, 31.30)]were superior to the control group in reducing TG value, the details will be shown in Table 4. The probability ranking of the different exercise interventions in terms of reducing TG value was ranked first by resistance training in the SUCRA for (SUCRA: 100% as shown in Fig. 4C).

**Table 4:** League table on TG.

Aerobic exercise	CON	Concurrent training	Resistance training
Aerobic exercise	0.02 (− 0.16, 0.20)	− 0.26 (− 0.52, 0.01)	− 23.97 (− 31.30, − 16.63)
− 0.02 (− 0.20, 0.16)	CON	− 0.28 (− 0.47, − 0.08)	− 23.99 (− 31.32, − 16.65)
0.26 (− 0.01, 0.52)	0.28 (0.08, 0.47)	Concurrent training	− 23.71 (− 31.05, − 16.37)
23.97 (16.63, 31.30)	23.99 (16.65, 31.32)	23.71 (16.37, 31.05)	Resistance training

#### High density lipoprotein (HDL)

All *P*-values for indirect and direct comparisons between all studies were tested for consistency and inconsistency, and all *P*-values were greater than 0.05, indicating that the effect of consistency between studies was acceptable. Details will be shown in Fig. 5B.

The results of the Network meta-analysis showed that relative to the control group's routine measures, concurrent training[MD = − 7.95, 95% CI (− 11.11, − 4.78)], aerobic exercise [MD = − 7.43, 95% CI (− 10.69, − 4.16)], resistance training [MD = 7.95, 95% CI (4.78, 11.11)], were superior to the control group in improving HDL value, the details will be shown in Table 5. The probability ranking of the different exercise interventions in terms of improving HDL value was ranked first by resistance training in the SUCRA for (SUCRA: 100% as shown in Fig. 5C).

**Table 5:** League table on HDL.

Aerobic exercise	CON	Concurrent training	Resistance training
Aerobic exercise	− 1.24 (− 2.36, − 0.12)	− 0.52 (− 2.05, 1.01)	7.43 (4.16, 10.69)
1.24 (0.12, 2.36)	CON	0.72 (− 0.41, 1.85)	8.67 (5.74, 11.60)
0.52 (− 1.01, 2.05)	− 0.72 (− 1.85, 0.41)	Concurrent training	7.95 (4.78, 11.11)
− 7.43 (− 10.69, − 4.16)	− 8.67 (− 11.60, − 5.74)	− 7.95 (− 11.11, − 4.78)	Resistance training

#### Low density lipoprotein (LDL)

Every P-value from indirect and direct comparisons across all studies was scrutinized for both consistency and inconsistency. All P-values turned out to be more than 0.05, pointing to a commendable level of coherence among the studies. More comprehensive details will be presented in Fig. 6B.

The results of the Network meta-analysis showed that relative to the control group's routine measures, resistance training [MD = − 32.95, 95% CI (− 52.06, − 13.86)], aerobic exercise [MD = 29.67, 95% CI (16.22, 43.13)], concurrent training [MD = 32.95, 95% CI (13.86, 52.06)], were superior to the control group in reducing LDL value, the details will be shown in Table 6. The probability ranking of the different exercise interventions in terms of reducing LDL value was ranked first by resistance training in the SUCRA for (SUCRA: 100% as shown in Fig. 6C).

**Table 6:** League table on LDL.

Aerobic exercise	CON	Concurrent training	Resistance training
Aerobic exercise	3.76 (− 1.45, 8.96)	3.28 (− 2.36, 8.93)	− 29.67 (− 43.13, − 16.22)
− 3.76 (− 8.96, 1.45)	CON	− 0.48 (− 11.32, 10.38)	− 33.43 (− 52.09, − 14.77)
− 3.28 (− 8.93, 2.36)	0.48 (− 10.38, 11.32)	Concurrent training	− 32.95 (− 52.06, − 13.86)
29.67 (16.22, 43.13)	33.43 (14.77, 52.09)	32.95 (13.86, 52.06)	Resistance training

#### Systolic pressure (SBP)

All P-values stemming from both indirect and direct comparisons among every study were assessed for uniformity and disparity. All of these P-values exceeded the 0.05 threshold, signaling an acceptable level of consistency among the studies. Further elaborations will be depicted in Fig. 7B.

The results of the Network meta-analysis showed that relative to the control group's routine measures, resistance training [MD = − 4.47, 95% CI (− 11.84, 2.90)], concurrent training [MD = 4.19, 95% CI (− 2.78, 11.16)], aerobic exercise [MD = 4.76, 95% CI (− 2.21, 11.73)] were superior to the control group in reducing SBP value, the details will be shown in Table 7. The probability ranking of the different exercise interventions in terms of reducing SBP value was ranked first by resistance training in the SUCRA for (SUCRA: 92.5% as shown in Fig. 7C).

**Table 7:** League table on SBP.

Aerobic exercise	CON	Concurrent training	Resistance training
Aerobic exercise	2.18 (− 0.91, 5.28)	− 0.57 (− 4.85, 3.71)	− 4.76 (− 11.73, 2.21)
− 2.18 (− 5.28, 0.91)	CON	− 2.75 (− 5.84, 0.33)	− 6.94 (− 13.20, − 0.69)
0.57 (− 3.71, 4.85)	2.75 (− 0.33, 5.84)	Concurrent training	− 4.19 (− 11.16, 2.78)
4.76 (− 2.21, 11.73)	6.94 (0.69, 13.20)	4.19 (− 2.78, 11.16)	Resistance training

#### Diastolic pressure (DBP)

All *P*-values for indirect and direct comparisons between all studies were tested for consistency and inconsistency, and all *P*-values were greater than 0.05, indicating that the effect of consistency between studies was acceptable. Details will be shown in Fig. 8B.

The results of the Network meta-analysis showed that relative to the control group's routine measures, concurrent training [MD = − 0.05, 95% CI (− 7.50, 7.40)], resistance training [MD = 0.05, 95% CI (− 7.40, 7.50)], aerobic exercise [MD = 1.01, 95% CI (− 6.43, 8.44)] were superior to the control group in reducing DBP value, the details will be shown in Table 8. The probability ranking of the different exercise interventions in terms of reducing DBP value was ranked first by concurrent training in the SUCRA for (SUCRA: 71.2 % as shown in Fig. 8C).

**Table 8:** League table on DBP.

Aerobic exercise	CON	Concurrent training	Resistance training
Aerobic exercise	1.56 (− 1.44, 4.55)	− 1.06 (− 5.23, 3.11)	− 1.01 (− 8.44, 6.43)
− 1.56 (− 4.55, 1.44)	CON	− 2.61 (− 5.65, 0.42)	− 2.56 (− 9.36, 4.24)
1.06 (− 3.11, 5.23)	2.61 (− 0.42, 5.65)	Concurrent training	0.05 (− 7.40, 7.50)
1.01 (− 6.43,8.44)	2.56 (− 4.24, 9.36)	− 0.05 (− 7.50, 7.40)	Resistance training

#### Glucose (Glu)

P-values associated with indirect and direct comparisons across all studies were examined for their consistency and inconsistency. Each of these P-values surpassed 0.05, which underscores an acceptable degree of harmony among the various studies. Additional particulars will be illustrated in Fig. 9B

The results of the Network meta-analysis showed that relative to the control group's routine measures, concurrent training [MD = − 0.47, 95% CI (− 1.00, 0.05)], aerobic exercise [MD = − 0.40, 95% CI (− 0.89, 0.09)], were superior to the control group in reducing Glu value, the details will be shown in Table 9. The probability ranking of the different exercise interventions in terms of reducing Glu value was ranked first by concurrent training in the SUCRA for (SUCRA: 87.6% as shown in Fig. 9C).

**Table 9:** League table on Glu.

Aerobic exercise	CON	Concurrent training	Resistance training
Aerobic exercise	0.29 (0.06, 0.52)	− 0.08 (− 0.43, 0.27)	0.40 (− 0.09, 0.89)
− 0.29 (− 0.52, − 0.06)	CON	− 0.37 (− 0.63, − 0.10)	0.11 (− 0.34, 0.56)
0.08 (− 0.27, 0.43)	0.37 (0.10, 0.63)	Concurrent training	0.47 (− 0.05, 1.00)
− 0.40 (− 0.89, 0.09)	− 0.11 (− 0.56, 0.34)	− 0.47 (− 1.00, 0.05)	Resistance training

#### Insulin

Every P-value from both direct and indirect comparisons across all studies was subjected to tests for consistency and inconsistency. All of these P-values registered above 0.05, signifying an acceptable degree of uniformity across the studies. More extensive details will be portrayed in Fig. 10B.

The results of the Network meta-analysis showed that relative to the control group's routine measures, aerobic exercise [MD = − 2.34, 95% CI (− 5.61, 0.94)], concurrent training [MD = − 1.07, 95% CI (− 4.93, 2.80)], resistance training [MD = 1.07, 95% CI (− 2.80, 4.93)]were superior to the control group in reducing insulin value, , the details will be shown in Table 10. The probability ranking of the different exercise interventions in terms of reducing insulin value was ranked first by aerobic exercise in the SUCRA for (SUCRA: 89.1% as shown in Fig. 10C).

**Table 10:** League table on Insulin.

Aerobic exercise	CON	Concurrent training	Resistance training
Aerobic exercise	4.93 (2.66, 7.20)	1.27 (− 2.29, 4.83)	2.34 (− 0.94, 5.61)
− 4.93 (− 7.20, − 2.66)	CON	− 3.66 (− 6.41, − 0.91)	− 2.59 (− 5.31, 0.14)
− 1.27 (− 4.83, 2.29)	3.66 (0.91, 6.41)	Concurrent training	1.07 (− 2.80, 4.93)
− 2.34 (− 5.61, 0.94)	2.59 (− 0.14, 5.31)	− 1.07 (− 4.93, 2.80)	Resistance training

#### Homeostatic model assessment of insulin resistance (HOMA-IR)

All P-values, derived from both indirect and direct comparisons across all the studies, were inspected for consistency as well as inconsistency. All these P-values exceeded the threshold of 0.05, denoting an acceptable level of consistency amongst the studies. Further specifics will be demonstrated in Fig. 11B.

The results of the Network meta-analysis showed that relative to the control group's routine measures, concurrent training [MD = − 0.40, 95% CI (− 0.83, 0.03)], aerobic exercise [MD = − 0.19, 95% CI (− 0.54, 0.15)], resistance training [MD = 0.40, 95% CI (− 0.03, 0.83)] were superior to the control group in reducing HOMA-IR value, the details will be shown in Table 11. The probability ranking of the different exercise interventions in terms of reducing HOMA-IR value was ranked first by concurrent training in the SUCRA for (SUCRA:93.8 % as shown in Fig. 11C).

**Table 11:** League table on HOMA-IR.

Aerobic exercise	CON	Concurrent training	Resistance training
Aerobic exercise	0.61 (0.36, 0.86)	− 0.21 (− 0.61, 0.20)	0.19 (− 0.15, 0.54)
− 0.61 (− 0.86, − 0.36)	CON	− 0.82 (− 1.16, − 0.47)	− 0.42 (− 0.71, − 0.12)
0.21 (− 0.20, 0.61)	0.82 (0.47, 1.16)	Concurrent training	0.40 (− 0.03, 0.83)
− 0.19 (− 0.54, 0.15)	0.42 (0.12, 0.71)	− 0.40 (− 0.83, 0.03)	Resistance training

#### Hemoglobin A1c (Hba1c)

**Figure 12:**
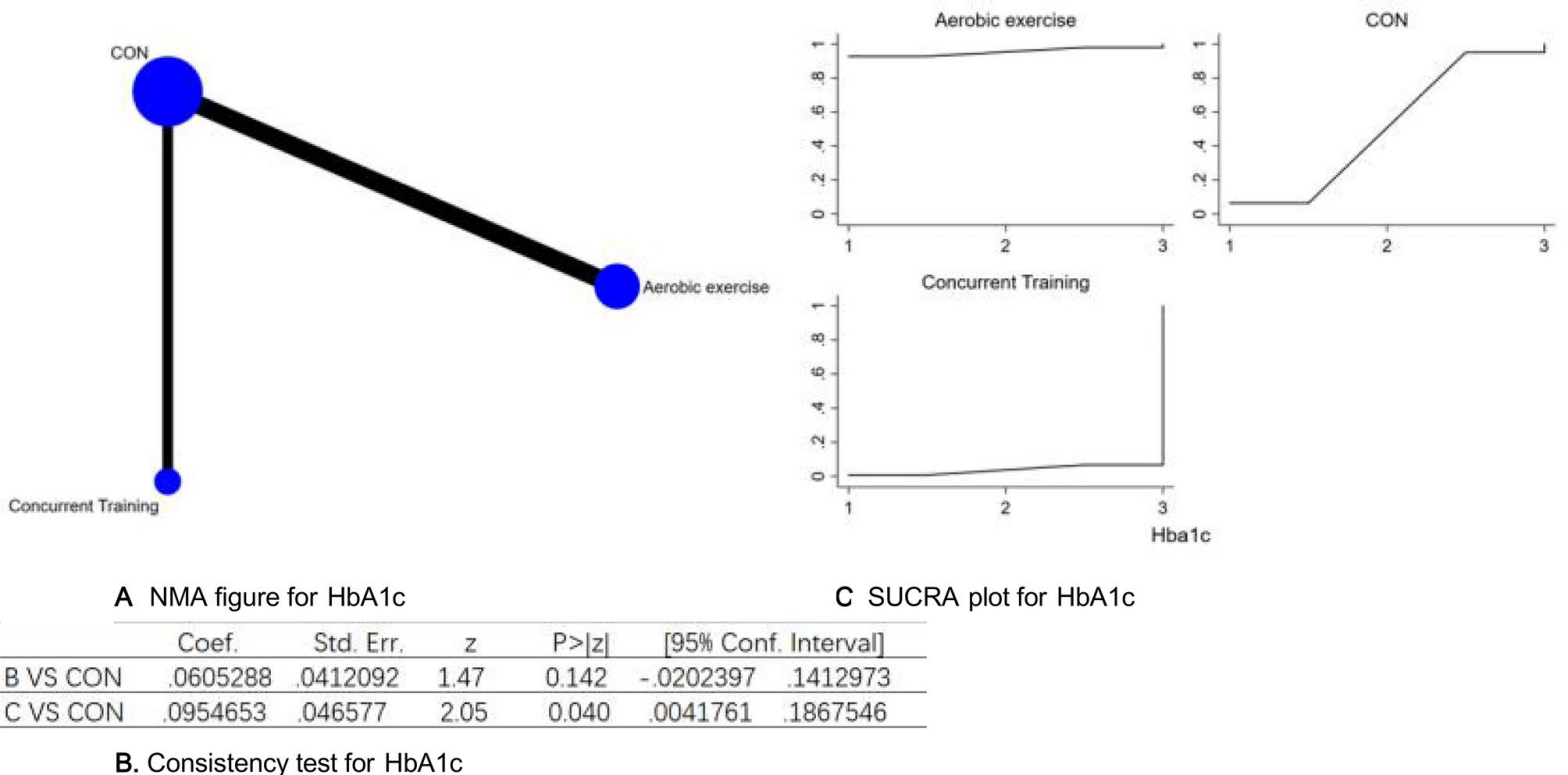
(**A**) NMA figure for HbA1c, (**B**) Consistency test for HbA1c, (**C**) SUCRA plot for HbA1c.

Each P-value from indirect and direct comparisons involving all studies was evaluated for uniformity and variance. Every P-value was found to be above 0.05, which suggests a satisfactory level of agreement within the studies. More specifics will be outlined in Fig. 12B.

The results of the Network meta-analysis showed that relative to the control group's routine measures, aerobic exercise [MD = − 0.10 (− 0.19, − 0.00), 95% CI (− 0.19,0.00)] were superior to the control group in reducing Hba1c value, the details will be shown in Table 12. The probability ranking of the different exercise interventions in terms of reducing Hba1c value was ranked first by aerobic exercise in the SUCRA for (SUCRA:95.3 % as shown in Fig. 12C).

**Table 12:** League table on HbA1c.

Aerobic exercise	CON	Concurrent Training
Aerobic exercise	0.06 (− 0.02, 0.14)	0.10 (0.00, 0.19)
− 0.06 (− 0.14, 0.02)	CON	0.03 (− 0.01, 0.08)
− 0.10 (− 0.19, − 0.00)	− 0.03 (− 0.08, 0.01)	Concurrent training

### Publication bias test

**Figure 13:**
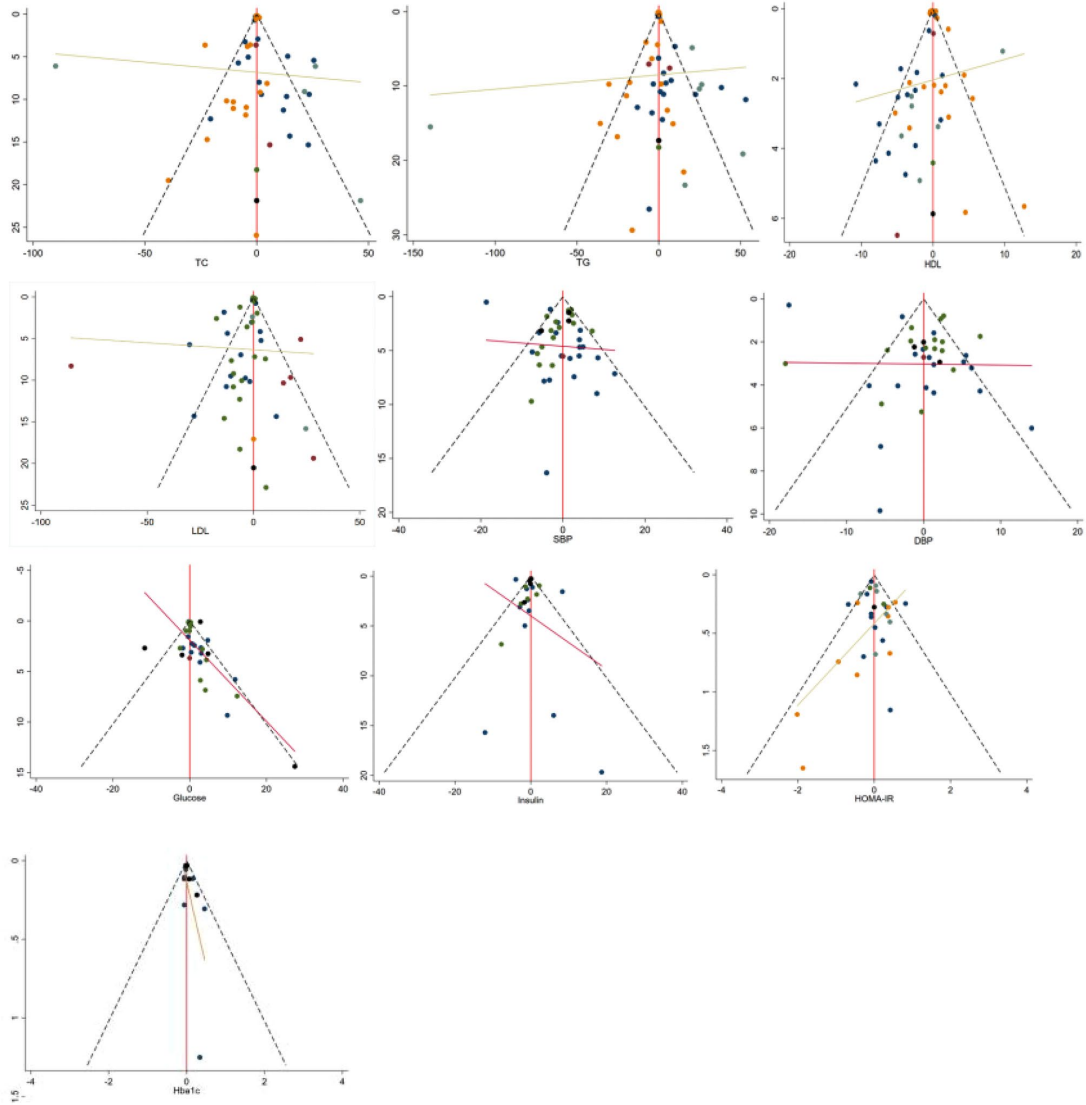
Funnel plot on publication bias.

We constructed separate funnel plots for all outcome indicators to test for possible publication bias. After visually examining the funnel plot did not reveal any significant publication bias^55^.Details as shown in Fig. 13.

## Discussion

In this study, we investigated the effects of various exercise interventions on the levels of abnormal intermediate disease biomarkers in populations with overweight and obese. A total of 56 studies, including 14 different exercise programs and 3193 people with obese and overweight, were included in the analysis, resulting in a substantial sample size. Our research findings suggest that resistance training can significantly improve dyslipidemia, reduce systolic blood pressure, while concurrent training can significantly reduce diastolic blood pressure and blood glucose levels. Aerobic exercise can significantly reduce blood glucose levels in obese and overweight individuals. Aerobic exercise is considered the most effective exercise intervention for reducing glycated hemoglobin and insulin levels, resistance training is the most effective exercise intervention for lowering systolic blood pressure and blood lipids, and concurrent training is the most effective exercise intervention for regulating diastolic blood pressure, reducing blood glucose and insulin resistance.

Numerous previous studies have shown that intermediate disease biomarker levels are abnormal in populations with overweight and obese^56–59^. The intermediate disease biomarkers discussed in this article reflect people' blood lipid, blood glucose, blood pressure levels. These markers are closely related to the metabolic and cardiovascular status of people, as well as the development of chronic diseases. Therefore, by analyzing these intermediate disease biomarkers, healthcare providers can improve people' overall health status and reduce the risk of chronic diseases based on the exercise intervention recommendations provided herein.

### Aerobic exercise

Aerobic exercise is widely regarded as the most effective method for weight loss in obese individuals ,this form of exercise, which involves continuous physical activity leading to increased heart rate and respiration, not only enhances strength and endurance but also stimulates metabolism, reduces fat storage, and promotes cardiovascular health, thereby contributing to overall well-being^60,61^.

The outcomes of our network meta-analysis revealed that compared to no exercise, aerobic exercise significantly managed blood lipid levels, blood pressure, and blood glucose in overweight and obese individuals. Moreover, among various exercise modalities, aerobic exercise stood out as the most effective method in lowering insulin and glycated hemoglobin concentrations within this population.

Clinicians can tailor aerobic exercise prescriptions to people with overweight and obese based on the results of this study^18–34^, and can choose appropriate forms of exercise from dance, walking, endurance training, cycling, running, and MICT. It is recommended that the duration of each exercise range from 30 to 90 mins, with a frequency of three to six times a week. Exercise intensity should be tailored to the patient's specific needs in order to effectively lower their blood glucose levels. Regular aerobic exercise can improve metabolic and physiological functions, alleviate physical strain, and boost overall health status.

### Resistance training

Our research findings reveal that all exercise types can effectively lower people' abnormal blood lipid levels when compared to sedentary control groups. Nevertheless, resistance training stands out as the most successful exercise type within this study. Resistance training not only has the ability to regulate blood lipid levels but can also decrease people' systolic blood pressure. It is crucial to highlight that, when compared to the non-exercising experimental control group, resistance training demonstrates less efficacy in reducing blood glucose levels for individuals with obese and overweight.

Based on our literature review^35–43^, clinicians may consider prescribing traditional exercises such as squats, lunges, bicep curls, push-ups, and shoulder presses. Each exercise should be executed with 8–15 repetitions per set and 2–6 sets per training session. In addition, alternative and engaging resistance training techniques, like rope training and whole-body vibration training, can be taken into account to boost people' enthusiasm, interest, and overall exercise performance.

Exercise sessions should span between 20 and 60 min, with warm-up and stretching exercises recommended before and after each session. A frequency of three or four times per week is advised, and exercise intensity should be tailored to suit the individual's unique needs.

### Concurrent training

Concurrent training (CT) is a comprehensive exercise method that combines aerobic and resistance training. Compared to inactivity, CT demonstrates significant effectiveness in adjusting blood glucose, HOMA-IR, and diastolic blood pressure in overweight and obese individuals. However, it has a limited impact on glycated hemoglobin levels. Therefore, it is not recommended to choose CT as the exercise approach when glycated hemoglobin, an intermediate disease marker, is abnormal in individuals with overweight and obese.

Therefore, we recommend that clinicians use CT as the main mode of exercise in their prescriptions for people with overweight and obese with significant abnormalities in diastolic blood pressure, blood glucose, and insulin resistance markers.

Clinicians can incorporate interval training, karate, HICT, or HIEX as exercise forms in their prescriptions, scheduling three to five training sessions per week, each lasting between 11 and 60 mins^23,29,34,44–53^. The specific exercise intensity should be customized based on each individual's particular needs.

In summary, our study bears clinical significance in two key respects: firstly, it provides a theoretical foundation for the fact that exercise can significantly mitigate abnormal intermediate disease markers in people with overweight and obese; secondly, it allows clinicians to integrate our findings with people' abnormal intermediate disease markers to tailor exercise prescriptions, thereby fostering overall patient health.

## Strengths and limitations

Our study encompasses 56 research projects and 3193 people, which constitutes a substantial sample size, and offers a comprehensive and updated review of exercise interventions in people with overweight and obese. We've integrated three innovative interventions—water sports, whole—body vibration training, and rope training—to compare with other interventions, thereby delivering the most recent and exhaustive evidence-based recommendations.

However, our study shares some limitations with the research it is based on. Despite our efforts to control study heterogeneity, some heterogeneity is inevitable between studies, such as the proportion of male and female participants and regional variations. Readers should interpret our findings cautiously due to the strict literature screening criteria, the limited number of included studies, and limited direct comparison evidence for certain interventions. This highlights the need for further expansion of relevant research.

Lastly, our study only included 14 exercise interventions that met the literature screening criteria, but in reality, there are many more interventions that can positively impact the intermediate markers of individuals with overweight and obese. Future research can broaden the scope by including more types of exercise interventions, offering more choices for different people; analyze the response differences among different populations (such as age, gender, race, and cultural background) to exercise interventions, to develop more personalized exercise plans; and explore the long-term effects of exercise interventions to better assess their sustainability in improving the health of overweight and obese people.

In summary, although our study has some limitations, it still provides valuable information on exercise interventions for overweight and obese people. By analyzing various types of exercise interventions, we offer evidence-based recommendations for healthcare providers on how to develop more effective treatment strategies. In the future, we hope to see more research on exercise interventions to better meet the needs of different people and help them achieve healthier lifestyles.

## Conclusions

The study hints at potential mechanisms by which resistance, aerobic, and concurrent training could influence improvements in health markers for individuals with overweight and obesity, thus shedding light on the varying impacts of different exercise modalities on health outcomes. Resistance training enhances lean muscle mass, leading to an increase in metabolic rate and fat oxidation, improvement in lipid profiles , and reduction in systolic blood pressure. Conversely, aerobic training primarily improves insulin sensitivity and cardiovascular health while lowering blood glucose levels but may not significantly enhance muscle strength. Concurrent training combines the advantages of both exercise types by addressing diastolic blood pressure and blood glucose levels despite potential interference effects that could impact optimal strength or endurance gains.

Each modality offers distinct advantages: resistance training is optimal for enhancing muscle strength and lipid profiles, aerobic training excels in providing cardiovascular benefits and managing glucose levels, while concurrent training provides a comprehensive approach to simultaneously address multiple health markers. However, the selection of exercise should be tailored to individual health objectives, preferences, and specific conditions while acknowledging the limitations and strengths associated with each modality (Supplementary Information).

Based on the collective insights derived from this study, it is recommended that clinicians adopt a personalized exercise prescription approach when working with overweight and obese populations. By aligning exercise modalities with individual health profiles and objectives, clinicians can develop targeted interventions that not only enhance overall health but also mitigate the risk of chronic conditions. This approach capitalizes on the distinct advantages offered by resistance, aerobic, and concurrent training to cater to diverse health needs.

### Supplementary Information

Supplementary Information.

## Data Availability

The data that support the findings of the study are available from the first author, upon reasonable request.
