# Supplementary material for: Evaluating the impact of exercise on intermediate disease markers in overweight and obese individuals through a network meta-analysis of randomized controlled trials
Source: Sci Rep. 2024 May 27;14:12137. doi: 10.1038/s41598-024-62677-w (PMC11130208; doi:10.1038/s41598-024-62677-w)
Supplement: Supplementary file 1 — Supplementary Information. [file 41598_2024_62677_MOESM1_ESM.pdf]

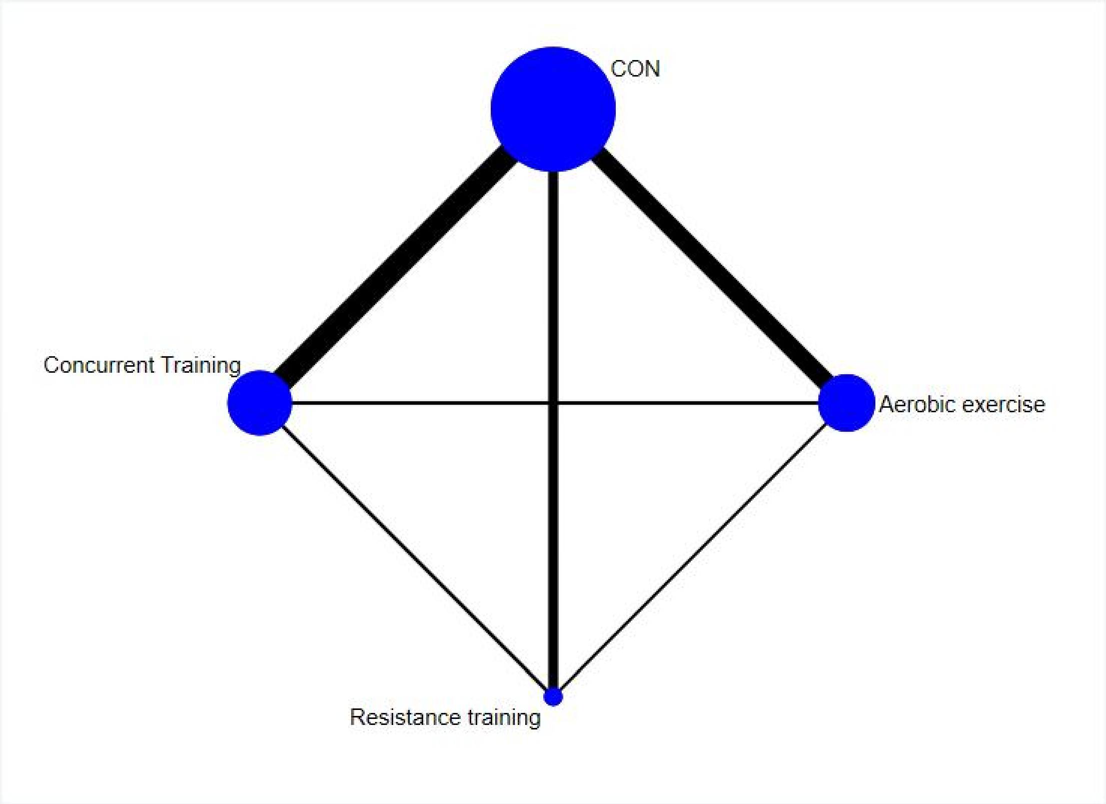

B VS CON	3.755894	2.656973	1.41	0.157	-1.451678	8.963466
C VS CON	3.284384	2.883881	1.14	0.255	-2.367918	8.936686
D VS CON	-29.67407	6.863758	-4.32	0.000	-43.12679	-16.22135

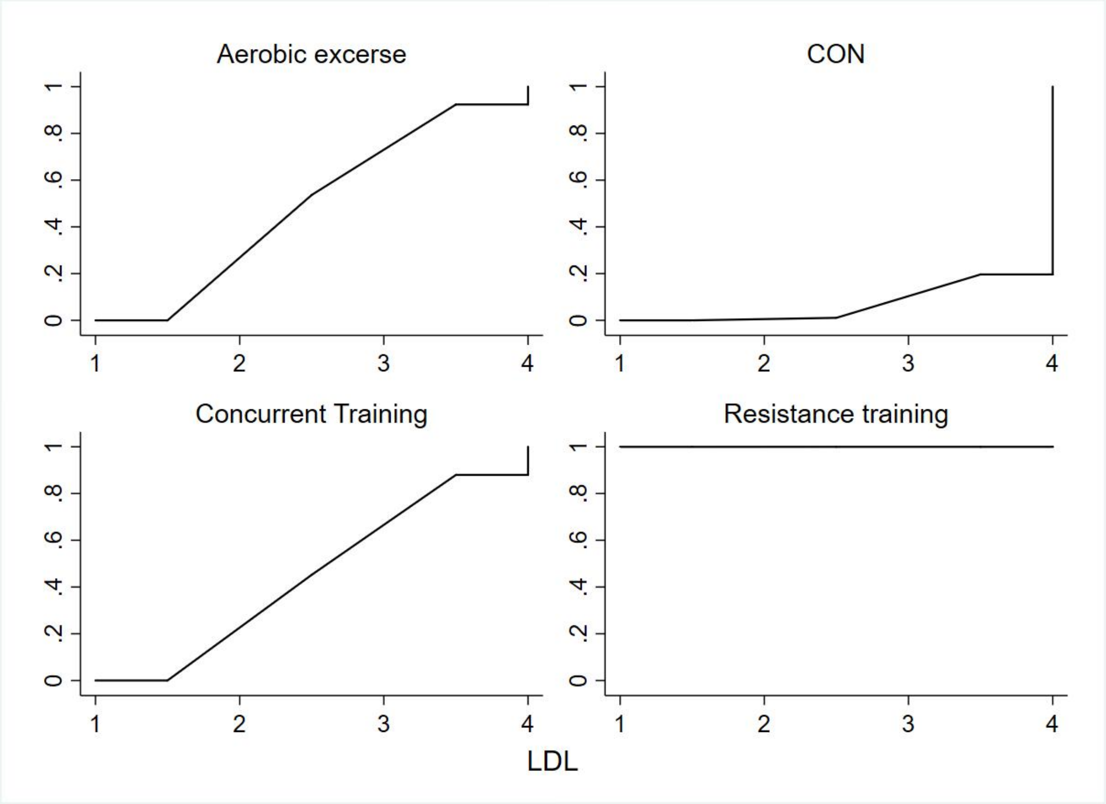

Aerobic exercise	_CON	_Concurrent Training_	_Resistance training_
Aerobic exercise	3.76 (-1.45,8.96)	3.28 (-2.36,8.93)	-29.67 (-43.13, -16.22)
-3.76 (-8.96,1.45)	CON	-0.48 (-11.32,10.38)	-33.43 (-52.09, -14.77)
-3.28 (-8.93,2.36)	0.48 (-10.38,11.32)	Concurrent Training	-32.95 (-52.06, -13.86)
29.67 (16.22,43.13)	33.43 (14.77,52.09)	32.95 (13.86,52.06)	Resistance training

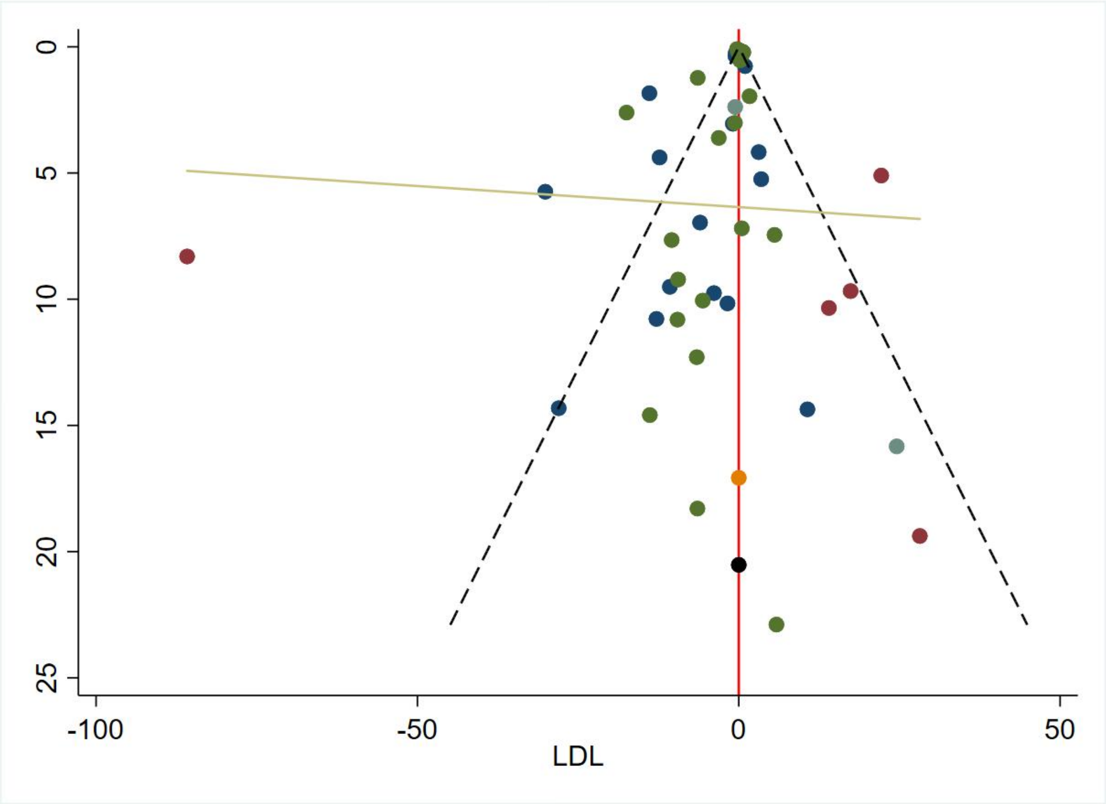

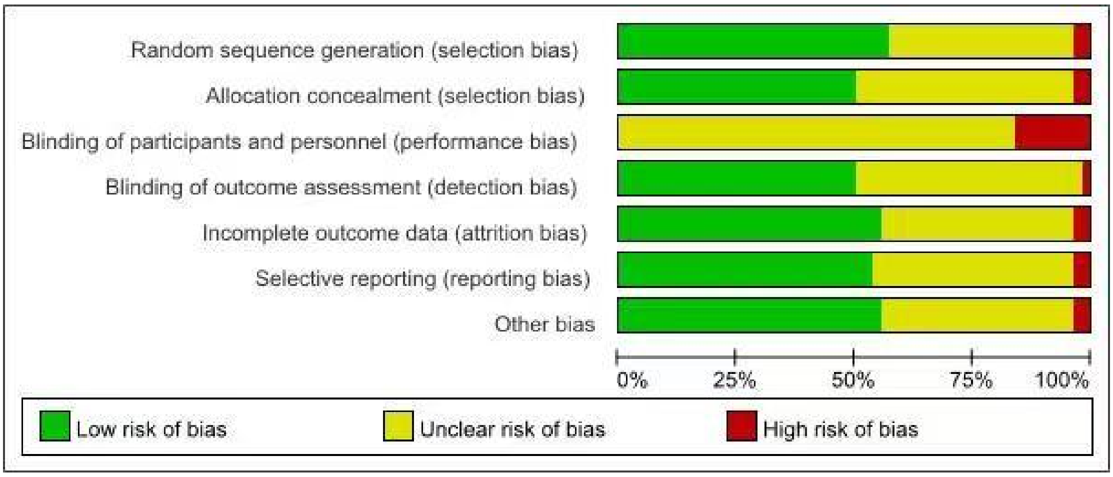

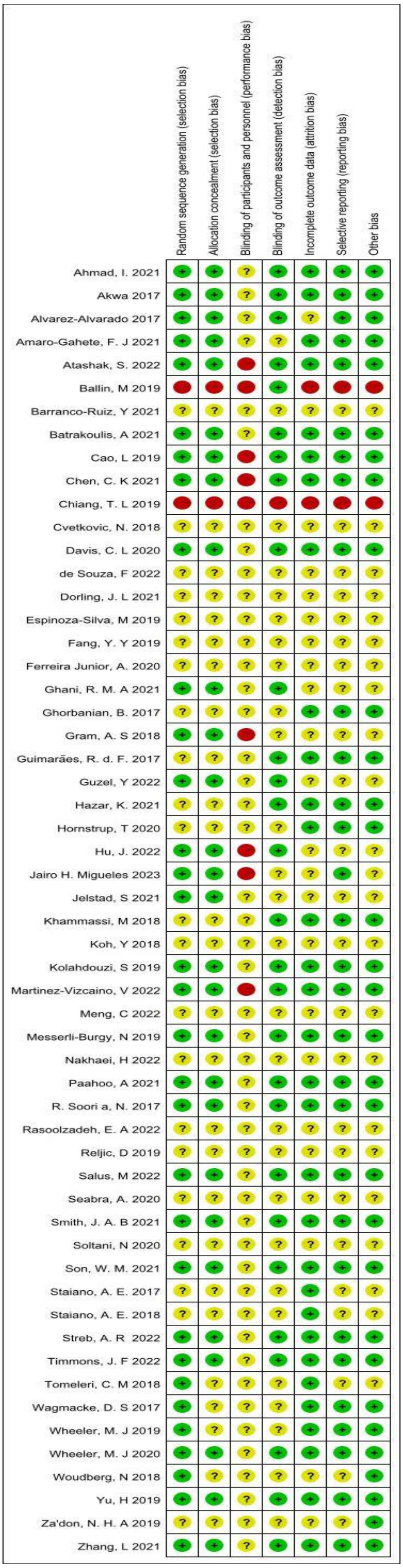
Random sequence generation (selection bias)
Allocation concealment (selection bias)
Blinding of participants and personnel (performance bias)
Blinding of outcome assessment (detection bias)
Incomplete outcome data (attrition bias)
Selective reporting (reporting bias)
Other bias
Ahmad, I. 2021
Akwa 2017
Alvarez-Alvarado 2017
Amaro-Gahete, F. J 2021
Atashak, S. 2022
Ballin, M 2019
Barranco-Ruiz, Y 2021
Batrakoulis, A 2021
Cao, L 2019
Chen, C. K 2021
Chiang, T. L 2019
Cvetkovic, N. 2018
Davis, C. L 2020
de Souza, F 2022
Dorling, J. L 2021
Espinoza-Silva, M 2019
Fang, Y. Y 2019
Ferreira Junior, A. 2020
Ghani, R. M. A 2021
Ghorbanian, B. 2017
Gram, A. S 2018
Guimarães, R. d. F. 2017
Guzel, Y 2022
Hazar, K. 2021
Hornstrup, T 2020
Hu, J. 2022
Jairo H. Migueles 2023
Jelstad, S 2021
Khammassi, M 2018
Koh, Y 2018
Kolahdouzi, S 2019
Martinez-Vizcaino, V 2022
Meng, C 2022
Messerli-Burgy, N 2019
Nakhaei, H 2022
Paahoo, A 2021
R. Soori a, N. 2017
Rasoolzadeh, E. A 2022
Reljic, D 2019
Salus, M 2022
Seabra, A. 2020
Smith, J. A. B 2021
Soltani, N 2020
Son, W. M. 2021
Staiano, A. E. 2017
Staiano, A. E. 2018
Streb, A. R 2022
Timmons, J. F 2022
Tomeleri, C. M 2018
Wagmacke, D. S 2017
Wheeler, M. J 2019
Wheeler, M. J 2020
Woudberg, N 2018
Yu, H 2019
Za'don, N. H. A 2019
Zhang, L 2021
